# Increasing polymer molecular weight enables low-donor-content, efficient and scalable semi-transparent organic solar cells

**DOI:** 10.1039/d5ta07234d

**Published:** 2026-01-27

**Authors:** Martín Martín-Ruiz, Paula Pinyol-Castillo, Xabier Rodríguez-Martínez, Jaime Martín, Ignasi Burgués-Ceballos, Mariano Campoy-Quiles

**Affiliations:** a Institute of Materials Science of Barcelona, ICMAB-CSIC Campus Universitat Autònoma de Barcelona 08193 Bellaterra Barcelona Spain mcampoy@icmab.es; b EURECAT Technology Centre of Catalonia Parc Científic i de la Innovació TecnoCampus 08302 Mataró Barcelona Spain; c Institute of Energy Technologies, Department of Chemical Engineering, Barcelona Research Center in Multiscale Science and Engineering, Universitat Politècnica de Catalunya Eduard Maristany 16 (EEBE) 08019 Barcelona Spain; d Universidade da Coruña, Campus Industrial de Ferrol, CITENI Campus de Esteiro S/N 15471 Ferrol A Coruña Spain; e Oportunius Program, Axencia Galega de Innovacion, Xunta de Galicia Eua Airas Nunes, S/N, 15702 Santiago de Compostela Spain

## Abstract

The advent of non-fullerene acceptors has enabled high efficiencies in organic photovoltaics (OPVs). The active layer of such devices typically consists of a narrow-bandgap molecular acceptor (A), with strong light absorption in the near-infrared region, combined with a polymer donor (D) that harvests visible photons. Reducing the donor content is a good strategy to increase transparency, but often leads to lower power conversion efficiencies (PCEs) due to loss in absorption efficiency and, importantly, a worsening of the electrical properties. Here, we tackle this compromise by investigating if the improved electrical properties granted by high polymer molecular weight (*M*_w_) can simultaneously result in a high PCE and high visible transparency, thus leading to efficient semitransparent OPVs. We investigate the polymer : non-fullerene blend PTB7-Th : IEICO-4F as a function of blend ratio for two polymer *M*_w_ values. We show that increasing the PTB7-Th *M*_w_ from 57 kDa to 125 kDa promotes a film morphology that enhances charge carrier mobility. Moreover, we demonstrate that using high-*M*_w_ PTB7-Th enables high PCEs in blends with as low a polymer content as 28%. Interestingly, we find that this behaviour can be explained by improved percolation (granted by higher *M*_w_) and higher acceptor crystallinity. In order to assess the scalability of the system, we compared devices fabricated in nitrogen or in air, and investigated the use of xylene as a greener solvent, the effect of increasing the cell area, the use of semitransparent electrodes and the fabrication of modules, identifying cell area as the most critical factor that negatively impacts PCE. Finally, we show the generality of the concept by extending it to two other polymers and two other acceptor molecules.

## Abstract 2

Increasing the donor’s molecular weight improves transport and enables high-efficiency solar cells with reduced donor content, which in turn improves transparency, leading to boosted semitransparent photovoltaics.

## Graphical abstract

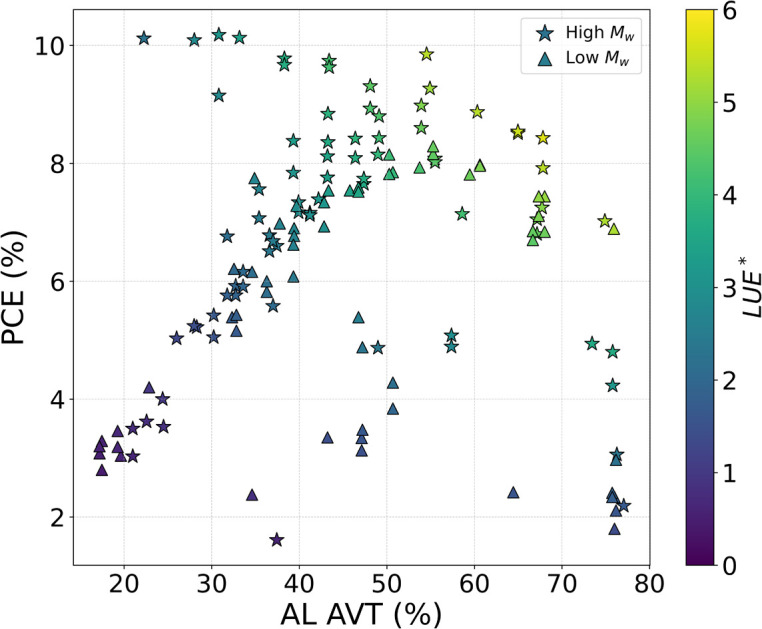

## 1 Introduction

Organic photovoltaics (OPVs) have emerged as a promising renewable energy technology due to their flexibility, lightweight nature, and potential for low-cost, large-area fabrication. Semitransparent organic photovoltaics (ST-OPVs), which allow visible light to (partially) pass through while converting sunlight into electrical energy, are particularly attractive for applications such as building-integrated photovoltaics (BIPVs),^1,2^ agrivoltaics,^3–7^ and power-generating windows,^8,9^ where transparency is of high importance.^10^ Their inherent flexibility, tunable transparency, and compatibility with low-cost, large-area fabrication techniques make them a promising alternative to conventional solar cells. Numerous studies have demonstrated the successful incorporation of ST-OPVs into architectural elements, underscoring their ability to generate renewable energy while preserving aesthetics and transparency. In the realm of agrivoltaics, several research groups have highlighted how ST-OPVs can enable simultaneous cultivation of crops and energy generation, thus optimizing land use and the overall efficiency of the system.^3–7^ Furthermore, in the field of photovoltaic windows, Xueting Wu *et al.*^8^ reported devices achieving a power conversion efficiency (PCE) exceeding 9% with an average visible transmittance (AVT) above 40%, illustrating the feasibility of ST-OPVs for transparent applications.

Several strategies have been developed to enhance the transparency of OPVs, focusing on both device architecture and materials. On the architecture side, one of the most critical layers in semitransparent devices is the electrode, which must allow light to pass through while maintaining a high electrical conductivity. A wide range of transparent-electrode technologies have been explored, including ultrathin bilayer metal films,^11,12^ transparent conductive oxides, metal nanowires,^13,14^ transparent conductive polymers,^15^ carbon nanotubes, and graphene.^16^ Increasing transmission or IR absorption employing photonic structures has also been widely explored. In terms of materials, besides synthesising materials with an enhanced transparency window in the visible range, other approaches have been used for improved efficiency and stability, such as ternary compound strategies,^17,18^ layer-by-layer deposition,^19^ bifacial illumination,^20^ and donor/acceptor (D/A) ratio optimization.^21–24^

To achieve high transparency in OPVs, the use of narrow-bandgap materials that absorb predominantly in the near-infrared region is critical. In general, acceptor materials in donor–acceptor systems absorb light in the near-infrared (NIR) region, helping to preserve transparency in the visible spectrum. In contrast, donor materials typically show stronger absorption in the visible range and limited absorption in the NIR, which can compromise the overall transparency of the device. Considering the stronger absorption of the donor compound in the visible range, reducing its content is a sound strategy to increase the transparency of the active layer. However, an excessively low donor concentration can result in suboptimal morphology and phase separation, leading to increased charge recombination losses^21^ and a reduced PCE. In this work, PTB7-Th and IEICO-4F were employed due to their complementary absorption properties and good photovoltaic performance. PTB7-Th absorbs mainly in the visible region, with an absorption peak around 700 nm, while IEICO-4F exhibits strong light harvesting in the near-infrared (NIR). This material combination has been widely reported to achieve a good balance between PCE and AVT in ST-OPVs.^21–26^

On the other hand, numerous studies have investigated the effect of polymer molecular weight (*M*_w_) on organic solar-cell performance. It has been demonstrated that higher-molecular-weight donor polymers tend to exhibit enhanced chain entanglement, improved film morphology, and more efficient charge carrier transport, all of which contribute to higher device performance. For example, Intemann *et al.* reported that increasing the molecular weight of the donor polymer can result in improved film absorption and increased charge carrier mobilities, yielding enhanced photovoltaic performance.^27^ Similarly, Riera-Galindo *et al.* showed that high-molecular-weight polymers can significantly improve charge mobility and stability,^28^ enabling thickness-resilient performance. Ding *et al.* highlighted that efficient solar cells are more stable when higher-molecular-weight polymers are used, demonstrating an improvement in PCE with increasing *M*_w_, mainly due to enhancements in short-circuit current (*J*_sc_).^29^ Furthermore, Tran *et al.* have shown that increasing the number-average molecular weight (*M*_n_) up to an optimal value of 55 kDa correlates with optimal charge transport and morphology and a constant power conversion efficiency (PCE) across varying active-layer thicknesses,^30^ a finding that is highly promising for upscaling.

Here, we present the systematic optimization of the D/A ratio coupled with molecular-weight tuning of PTB7-Th, aiming at improving transparency without compromising solar-cell performance. By reducing the donor content, we increase the visible transparency of the active layer. However, an excessively low donor concentration can result in suboptimal morphology and increased charge recombination losses.^21^ To address this limitation, we compared devices fabricated with low-molecular-weight (57 kDa) and high-molecular-weight (125 kDa) PTB7-Th. This dual strategy, optimizing the D/A ratio to balance optical and electrical properties while leveraging higher molecular weight to enhance charge mobility and improve the *J*–*V* characteristics, demonstrates a promising pathway toward the development of high-performance semitransparent organic solar cells. In order to show the generality of the concept, we made solar cells with four additional donor : acceptor systems and found that low donor blends typically work better for high-molecular-weight polymers, either in absolute terms, or in terms of thickness resilience. For selected systems, we complement the device data with mobility measurements as well as spectroscopic and GIWAXS data. In the second part of the paper, we evaluate the scalability of the high-*M*_w_-based devices by fabricating solar cells in air rather than inside a nitrogen-filled glovebox, replacing chlorobenzene with a greener solvent (*o*-xylene), increasing the cell area, replacing the top electrode with a semitransparent electrode, and fabricating modules with series-connected cells.

## 2 Results and discussion

**Fig. 1:**
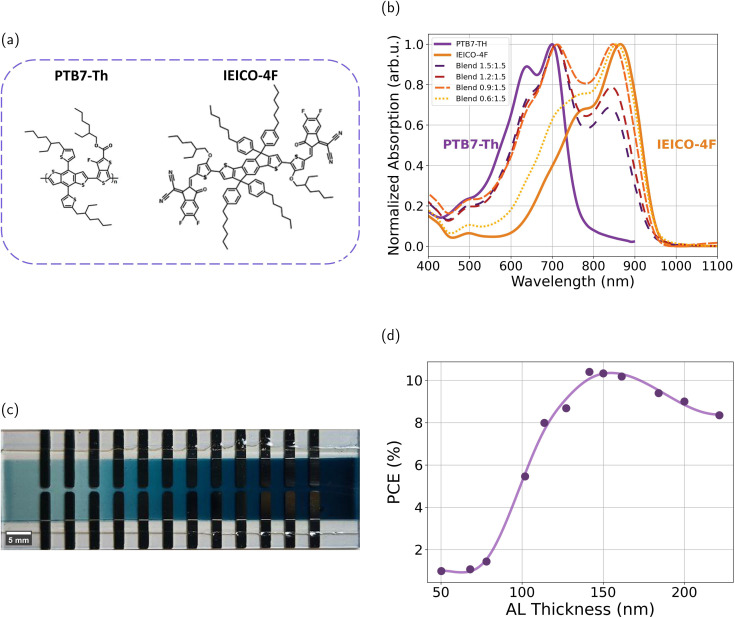
(a) Chemical structures of the organic semiconductors PTB7-Th (donor) and IEICO-4F (acceptor). (b) Normalized absorption spectra of both PTB7-Th and IEICO-4F and blends with different D/A ratios. (c) Photograph of a sample with 24 pixels and a thickness gradient. (d) Power conversion efficiency (%) as a function of active-layer thickness.

The selection of PTB7-Th as the donor material and IEICO-4F as the non-fullerene acceptor was driven by the goal of optimizing both the power conversion efficiency (PCE) and light utilization properties. Their chemical structures are shown in Fig. 1a. The normalized absorption spectra of these materials (Fig. 1b) confirm that PTB7-Th mainly absorbs in the visible region (400–750 nm), with the 0–0 peak centered at 720 nm. IEICO-4F exhibits strong photon harvesting in the near-infrared spectral range, with the 0–0 peak centered at 865 nm. This combination, with complementary absorption, ensures efficient solar-spectrum utilization and photon harvesting of PTB7-Th : IEICO-4F blend films.^21,25^

In addition, this donor/acceptor combination is well established in the literature for semitransparent organic photovoltaic (OPV) applications. In our laboratory, the PTB7-Th : IEICO-4F system has consistently exhibited higher power conversion efficiencies compared to PTB7-Th blends with alternative acceptors.^31^ In the experiments conducted to test the generality of the process, we again observed this trend (Table 1). IEICO-4F also features more extended near-infrared absorption relative to commonly used acceptors such as Y6, resulting in enhanced spectral complementarity with PTB7-Th. These characteristics make the PTB7-Th : IEICO-4F system a well-suited model for investigating the impact of donor molecular weight on the optoelectronic properties of semitransparent OPVs.

Active-layer thickness is one of the key parameters affecting both PCE and average visible transmittance (AVT). To minimize the required amount of materials and accelerate the screening process, a previously developed high-throughput screening method has been used.^32–34^ Specifically, we deposited the active layer (AL) in thickness gradients *via* decelerating blade coating. Each substrate is designed with 12 distinct thickness variations. To ensure reproducibility, each pixel is replicated, resulting in a total of 24 devices per substrate. This high-throughput approach provides a systematic way to evaluate the influence of AL thickness on device performance. Fig. 1c shows a photograph of a sample and Fig. 1d shows an example of the performance of the 12 devices with different thicknesses on a single substrate, illustrating how PCE varies systematically across the thickness gradient. Fig. S1 in the SI shows the *J*–*V* curve of each device. In this study, the maximum PCE was achieved at an AL thickness of around 140 nm. A total of 1872 devices were fabricated under the different conditions, as explained below.

### 2.1 Donor/acceptor ratio optimization

Optimization of the donor–acceptor ratio is a crucial first step in organic solar-cell development. It affects the transparency, morphology, charge transport, and overall device performance. To explore this, we fabricated devices with PTB7-Th : IEICO-4F blends at different D/A ratios, ranging from 1.5 : 1.5 to 0.4 : 1.5 (wt/wt). The normalized and non-normalized absorption spectra of blend films with different D/A ratios are shown in Fig. 1b and S2, respectively. As expected, reducing the PTB7-Th content in the blends reduces the absorption in the visible region and increases the absorption in the near-infrared (NIR) range. This can facilitate the development of semitransparent organic solar cells, as the eye is only sensitive to the 400–700 nm range.

**Fig. 2:**
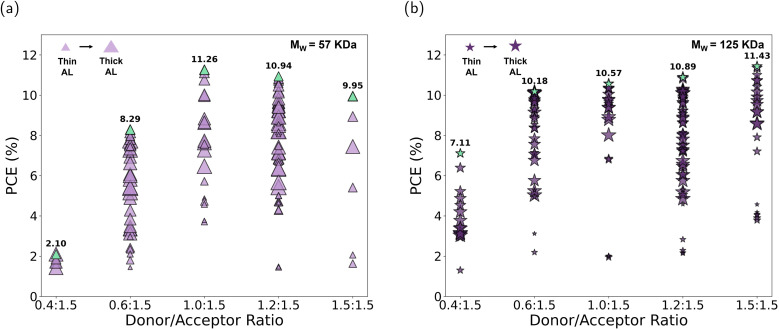
(a) Power conversion efficiency (%) and AL thickness (different-size symbols) of organic solar cells with PTB7-Th of 57 kDa molecular weight at various D/A ratios in weight. The data represents only the working devices for each combination, comprising around 253 devices, including the sample represented in Fig. 1d. (b) Comparison of power conversion efficiency (%) and AL thickness (different-size symbols) of organic solar cells with PTB7-Th of 125 kDa molecular weight at various D/A ratios in weight. Green symbols indicate the champion device fabricated for each configuration.

To observe the impact of these ratios on the photovoltaic (PV) performance, we fabricated devices with an inverted architecture: glass/ITO/ZnO/PTB7-Th : IEICO-4F/MoO_*x*_/Ag. The samples were prepared following the procedures outlined in the Experimental section for opaque devices, with a gradient in active-layer thickness. This resulted in devices with thicknesses varying from 70 nm to 300 nm, allowing for a comprehensive analysis of the performance as a function of AL thickness. The PCE results for devices based on 57 kDa *M*_w_ are presented in Fig. 2a, where the accumulation of data points at higher efficiencies indicates the optimal ratios. While the 1.0 : 1.5 ratio achieves the highest individual PCE values (reaching over 11%), it is the 1.2 : 1.5 ratio that demonstrates a stronger overall performance due to a higher density of points clustered in the top-efficiency range (9–10.5%). These results indicate that devices at the 1.2 : 1.5 ratio maintain consistently high efficiencies across a broader range of active-layer thicknesses, making this ratio more robust under varying fabrication conditions. At lower D/A ratios, such as 0.6 : 1.5, the PCE distribution shifts downward, with fewer points exceeding 8%, and decreases even more for the 0.4 : 1.5 D/A ratio. This trend is similar to that found by Hu *et al.*^21^ This decrease can be attributed to inadequate phase separation and suboptimal morphology, resulting in poorer charge transport pathways. In Fig. S4–S8 in the SI, the other PV parameters are shown. The trend observed for the fill factor (FF) closely follows that of the PCE. The optimum ratio is 1.2 : 1.5 with a notable accumulation of points above 60%. Beyond this point, the FF decreases significantly as the donor content is further reduced. The *J*_sc_ for this *M*_w_ of PTB7-Th increases as the donor content is reduced, reaching a maximum at 1.2 : 1.5 and decreasing considerably beyond this point. The open-circuit voltage (*V*_oc_) remains relatively stable across most tested ratios, slightly decreasing from 0.69 V to 0.67 V as the donor content is reduced. This trend can be attributed to increased charge-carrier recombination, or slightly modified energy levels due to microstructural changes.^35^ However, a more significant drop to less than 0.55 V is noted at a 0.4 : 1.5 ratio. The series resistance (*R*_s_) shows an increase at the 0.6 : 1.5 ratio, indicating that lower donor content leads to less efficient charge transport. The shunt resistance (*R*_sh_) reaches a maximum at the 1.2 : 1.5 ratio but decreases at polymer contents below 1.0 : 1.5. These findings underscore the limitation of using conventional *M*_w_ (57 kDa) PTB7-Th at reduced donor content. A reduction in donor concentration may enhance transparency, although it also results in decreased efficiency. Exploring the optimal D/A to form a suitable phase-separated structure is conducive to the generation and diffusion of excitons. The strong dependence of device performance on active-layer thickness and the poor performance at low D/A ratios highlight the need for alternative strategies to improve charge transport. Given these challenges observed with the 57 kDa *M*_w_ donor, we sought to explore whether higher-molecular-weight polymers could address these limitations, as already stated. The molecular weight of the polymer has an important effect on the aggregation tendency, phase separation, and main-chain orientation.^36^ The following section compares the performance of devices with PTB7-Th of 57 kDa and 125 kDa molecular weight to evaluate the impact on morphology, charge transport, and overall device efficiency.

### 2.2 Molecular-weight impact

#### 2.2.1 *J*–*V* characteristics

To investigate the influence of polymer molecular weight on device performance, we systematically fabricated solar cells using PTB7-Th with low (57 kDa) and high (125 kDa) molecular weight. Fig. 2b and S4–S8 from SI show that devices based on higher donor molecular weight outperform those with lower molecular weight at most donor/acceptor ratios tested. At higher D/A ratios (more than 1.0 : 1.5), the PCEs for medium- and high-*M*_w_ devices are comparable, with both showing a strong clustering of data points at high efficiencies. However, as the donor ratio decreases, the devices based on 125 kDa *M*_w_ maintain a higher density of points above 9% PCE, while devices based on 57 kDa *M*_w_ show a downward shift, with most thicknesses points falling below 8%. Interestingly, the large data set suggests that higher *M*_w_ leads to a smaller concentration dependence of the PCE with the optimum concentration at balanced ratios (1.5 : 1.5), while lower *M*_w_ leads to a sharper peak, maximized at higher acceptor contents.

The FF and *J*_sc_ trends highlight this difference. Higher-*M*_w_ devices maintain FFs above 60% even at the 0.6 : 1.5 ratio, with a noticeable accumulation of points above this percentage. In contrast, lower-*M*_w_ devices show a significant decrease in FF at the same ratio, with most points clustering below 50% for similar active-layer thicknesses. Devices with either 125 kDa or 57 kDa *M*_w_ show similar *J*_sc_ values for D/A ratios above 1.0 : 1.5, but lower-*M*_w_ devices exhibit more variability in *J*_sc_ for the same active-layer thickness range, especially at the 0.6 : 1.5 ratio. The same trend is observed with the open-circuit voltage (*V*_oc_) being more stable in devices with high molecular weight. These results suggest that charge extraction is more sensitive to morphological variations in blends with lower-molecular-weight polymers. The reduction in *R*_s_ for a low donor-ratio content suggests that the high-molecular-weight PTB7-Th supports more efficient charge transport pathways. The increase in *R*_sh_ indicates a reduced leakage current and better suppression of recombination pathways. The improvement in PCE observed can be mainly attributed to the differences in *J*_sc_ and FF. The absorption spectra, provided in the Fig. S3, are similar for both higher- and lower-molecular-weight samples, suggesting that differences in *J*_sc_ do not arise from differences in light absorption. According to prior findings, the increase in *M*_w_ may be linked to fewer recombination centers in the polymer, resulting in higher photocurrents.^29^ Additionally, higher *M*_w_ can be associated with a more favorable morphology, providing a larger donor/acceptor interface area and improved exciton dissociation efficiency, as well as better transport properties resulting from improved percolation.^28^

#### 2.2.2 Generality of the concepts

To determine whether the molecular-weight effect observed for PTB7-Th : IEICO-4F is specific to this system or extends to other donor/acceptor combinations, we examined three representative donor polymers (PTB7-Th, PTQ10 and PM6) paired with four non-fullerene acceptors (IEICO-4F, DTY6, COTIC-4F and Y6), using two donor/acceptor ratios (1.2 : 1.5 and 0.6 : 1.5), 12 different thicknesses and several donor molecular weights. Table 1 summarises the photovoltaic performance of the best pixel from each system, with the complete datasets included in the SI.

**Table 1:** Summary of the photovoltaic parameters (best pixel) for the additional donor/acceptor systems evaluated in the Generality of the concepts experiment, at two donor/acceptor ratios (1.2/1.5 and 0.6/1.5) and different donor molecular weights

Donor	Acceptor	Donor *M*_w_ (kDa)	Donor content	*V*_oc_ (V)	*J*_sc_ (mA cm^−2^)	FF (%)	PCE (%)
PTB7-Th	IEICO-4F	57.5	1.2	0.69	23.3	67.6	10.9
PTB7-Th	IEICO-4F	125.2	1.2	0.69	23.5	64.0	10.3
PTB7-Th	IEICO-4F	57.5	0.6	0.68	18.5	66.2	8.3
PTB7-Th	IEICO-4F	125.2	0.6	0.69	20.9	65.7	9.5
PTB7-Th	COTIC-4F	57.5	1.2	0.53	24.0	55.9	7.1
PTB7-Th	COTIC-4F	125.2	1.2	0.54	22.9	57.7	7.2
PTB7-Th	COTIC-4F	57.5	0.6	0.51	18.4	54	5.1
PTB7-Th	COTIC-4F	125.2	0.6	0.51	19.9	53.7	5.5
PTB7-Th	Y6	57.5	0.6	0.61	22.0	62.7	8.4
PTB7-Th	Y6	125.2	0.6	0.61	22.0	63.0	8.5
PTQ10	DTY6	63	1.2	0.88	14.0	46.5	5.8
PTQ10	DTY6	120	1.2	0.87	17.2	59.9	9.0
PTQ10	DTY6	147	1.2	0.87	16.9	63.70	9.4
PTQ10	DTY6	63	0.6	0.84	4.0	54.1	1.8
PTQ10	DTY6	120	0.6	0.85	10.6	65.3	5.9
PTQ10	DTY6	147	0.6	0.86	10.5	67.3	6.1
PM6	DTY6	83	1.2	0.84	25.8	76.3	16.7
PM6	DTY6	106	1.2	0.84	26.9	74.0	16.8
PM6	DTY6	83	0.6	0.84	23.2	74.4	14.6
PM6	DTY6	106	0.6	0.82	24.3	69.4	13.9

For the PTQ10 : DTY6 system, a clear dependence on donor molecular weight is observed, particularly when the donor fraction is reduced. Fig. S9 shows the correlation between PCE, FF and *J*_sc_ as a function of AL thickness (pixel) for three different *M*_w_ values (63, 120 and 147 kDa) and two D/A ratios (1.2 : 1.5 and 0.6 : 1.5). At the 1.2 donor ratio, the three molecular weights show comparable *V*_oc_ and *J*_sc_ values, although the lowest molecular weight exhibits noticeably lower FF in thicker pixels. When the donor ratio is decreased to 0.6, all molecular weights experience a reduction in *J*_sc_, but the effect is much stronger for the 63 kDa donor (70% loss) than for the samples based on 120 and 147 kDa donors (40% loss). In contrast, the FF increases for all *M*_w_ when lowering the donor ratio, consistent with the behaviour of PTB7-Th : IEICO-4F. These observations indicate that blends incorporating lower-*M*_w_ PTQ10 are more sensitive to donor dilution, whereas higher-*M*_w_ PTQ10 maintains more consistent device performance.

The PM6 : DTY6 devices exhibit a different behaviour. The two donor molecular weights investigated (83 and 106 kDa) are relatively close and high, resulting in only minor performance differences between the batches. When the donor ratio is reduced from 1.2 to 0.6, the *J*_sc_ decreases by approximately 10% for both molecular weights, while the fill factor (FF), already high at a 1.2 ratio, remains largely unaffected or shows only a slight decline. These results suggest that the PM6 : DTY6 blend morphology is less sensitive to donor dilution under these conditions, and that the molecular weight variation between batches is insufficient to induce significant changes in photovoltaic behaviour.

Two additional acceptors were evaluated with PTB7-Th. In the PTB7-Th : COTIC-4F system, both molecular weights exhibit comparable performance across donor ratios, and the reduction in *J*_sc_ with decreasing donor content is similar for both *M*_w_ values. For PTB7-Th : Y6, the two molecular weights tested at a 0.6 ratio yield nearly identical device performance, suggesting that the molecular weight impact in this system is again too small to induce a discernible trend. The corresponding evolution of PCE, FF, and *J*_sc_ as a function of AL thickness (nm) for both acceptor systems at a 0.6 : 1.5 D/A ratio is presented in the SI (Fig. S10). Optimal performance is comparable for both molecular weights when the AL thickness is approximately 100 nm. However, in the thicker region, devices with higher-*M*_w_ PTB7-Th exhibit slightly higher FF and *J*_sc_, indicating better transport properties. Taken together, these results demonstrate that the influence of donor molecular weight is not exclusive to PTB7-Th : IEICO-4F and can be observed in other donor/acceptor systems, particularly when the difference between molecular weight fractions is sufficiently large. We continue the subsequent analysis (stability, charge mobility, GIWAXS and transparency) using PTB7-Th : IEICO-4F as the representative system.

#### 2.2.3 Shelf lifetime analysis

While a full stability study is out of scope for the present manuscript, changing *M*_w_ can lead to different degrees of polymer entanglement, as well as slightly different glass transition temperatures, which in turn may affect morphological stability, as seen in shelf-lifetime or thermal-stability studies. In this work, all stability measurements were performed on encapsulated devices and under dark-storage conditions. Since our study focuses on how donor molecular weight and D/A ratio affect the intrinsic behaviour of the active-layer blend, it is essential to isolate these effects from extrinsic degradation pathways such as oxygen or moisture ingress, photo-oxidation or electrode instability. Encapsulation suppresses extrinsic degradation, while dark storage avoids light-driven chemical reactions.^37–39^

We performed a one year ISOS-D-1 dark-storage test on encapsulated devices. Devices were stored in the dark at room temperature and re-measured after 12 months. Fig. S11 and S12 show the evolution of PCE and *J*_sc_ as a function of the AL thickness (pixel) across the thickness gradient. Table S2 summarises the electrical parameters of the optimal pixel in each device. Across all four cases (1.2 and 0.6 donor ratios, higher- and lower-*M*_w_ PTB7-Th), the PCE decreases by 16.8–20.0% after one year. *V*_oc_ and *J*_sc_ vary only slightly, while FF undergoes moderate reductions (13.9–17.8%) for the optimal-thickness pixel. Importantly, the extent of the one-year change is similar for all devices. While the optimal pixels show only small changes in *J*_sc_, thicker pixels exhibit more pronounced losses, indicating that degradation increase at larger active-layer thicknesses. Interestingly, this trend is observed for all donor ratios and molecular weights, suggesting that the underlying ageing mechanism is intrinsic to the PTB7-Th : IEICO-4F system and device stack under dark-storage conditions and is not strongly dependent on the donor molecular weight or donor fraction. Overall, these results indicate that the one-year dark-storage degradation of PTB7-Th : IEICO-4F devices is modest and largely independent of molecular weight and donor ratio. Moreover, this small study also indicates that the variations in device performance observed throughout this work arise from differences in charge transport and morphology rather than from differences in stability.

#### 2.2.4 Charge mobility

We next evaluate the effect of molecular weight on charge transport within the photoactive layer by determining the charge carrier mobilities. Table 2 shows the electron (*µ*_e_) and hole (*µ*_h_) mobilities for devices made with low- and high-molecular-weight PTB7-Th at two distinct D/A ratios, as determined by space-charge-limited current (SCLC) measurements. Mobilities increase significantly with higher molecular weight both for 0.6 : 1.5 and 1.2 : 1.5 ratios. This improvement can be attributed to changes in microstructure that affect the interchain hopping rate and intra-chain transport.^28^ The hole mobility increases from 3.2 × 10^−4^ to 6.8 × 10^−4^ cm^2^ V^−1^ s^−1^ at the 1.2 : 1.5 D/A ratio and from 5.1 × 10^−4^ to 9.7 × 10^−4^ cm^2^ V^−1^ s^−1^ at the 0.6 : 1.5 D/A ratio. Electron mobilities also increase from the 1.2 : 1.5 to the 0.6 : 1.5 D/A ratios for both molecular weights. These results align with previous observations reported by Tran *et al.*, who found that increasing the polymer *M*_n_ from 21 kDa to 52 kDa (very similar to our case, where *M*_n_ changes from 23.78 kDa to 47.54 kDa) led to increases in both electron (*µ*_e_) and hole (*µ*_h_) mobilities.^30^

**Table 2:** Electron (*µ*_e_) and hole (*µ*_h_) mobilities for low- and high-molecular-weight PTB7-Th at different D/A ratios. Values were obtained from devices with 150–300-nm-thick active layers. Mobilities are averaged over 6 to 12 devices

*M*_w_	D/A ratio	*µ*_e_ (×10^−4^ cm^2^ V^−1^ s^−1^)	*µ*_h_ (×10^−4^ cm^2^ V ^−1^ s^−1^)
High	1.2 : 1.5	2.0 ± 0.9	6.8 ± 1.4
	0.6 : 1.5	2.8 ± 0.8	9.7 ± 1.1
Low	1.2 : 1.5	1.0 ± 0.6	3.2 ± 1.0
	0.6 : 1.5	2.9 ± 0.6	5.1 ± 0.5

If we consider the mobility ratio (*µ*_e_/*µ*_h_), one might conclude that a higher imbalance for the higher-*M*_w_ blend should lead to carrier accumulation and increased recombination. However, this interpretation might be suitable for systems with an approximately 1 : 1 donor/acceptor ratio, where balanced electron and hole mobilities are desirable as the volumetric pathways for both types of charges would also be similar. In our case, the blend is acceptor-dominated, and a more suitable descriptor could be the weighted mobility ratio (*µ*_e_*w*_A_)/(*µ*_h_*w*_D_), which accounts for both the carrier mobilities and the fractional content of donor and acceptor in the active layer. Using this definition, the higher-*M*_w_ blend yields a ratio of ≈0.72, whereas the lower-*M*_w_ blend gives a value of ≈1.42. The ratio for the higher *M*_w_ blend is therefore closer to the expected behaviour for efficient charge extraction in this type of asymmetric blend. Furthermore, in our inverted device structure, light impinges from the ITO/ZnO side, meaning that photogenerated charges will be closer to this electrode, and thus electrons need to travel less distance compared to holes. Under these conditions, a (*µ*_e_*w*_A_)/(*µ*_h_*w*_D_) < 1 would help to collect charges more efficiently and reduce electron accumulation. In the next section, we investigate the origin of these mobility differences by examining the molecular morphology using GIWAXS.

#### 2.2.5 Grazing incidence wide-angle X-ray scattering (GIWAXS)

We investigated the microstructure of neat PTB7-Th films of distinct molecular weight and their corresponding blends with IEICO-4F by means of GIWAXS measurements. All images and linecuts were acquired at a pitch angle of 0.12°, corrected for the real-time synchrotron beam flux, the integration time (1 second), the silicon background (20% subtraction) and the film thickness. Fig. S13a and d show the 2D GIWAXS images obtained after applying the Ewald's sphere correction for neat PTB7-Th films of high and low *M*_w_, respectively. In both cases, a qualitative inspection of the images indicates that these films have face-on texture. This is confirmed in the corresponding azimuthally-integrated linecuts (Fig. S13b and e), which signal a relatively broad reflection at *q* = 16.0 ± 0.2 nm^−1^ and *q* = 15.9 ± 0.3 nm^−1^ in the out-of-plane direction of the high- and low-*M*_w_ batches, respectively, here assigned to the π–π stacked planes along the [010] direction. There is also a sharper peak at *q* = 2.7 ± 0.1 nm^−1^, appearing predominantly in the in-plane linecuts that correspond to the lamellar reflections or (100) planes. Based on the corresponding coherence length (*L*_c_) and the paracrystallinity disorder parameter (*g*), the crystallites yielding the (010) and (100) reflections are of poor quality: *L*_c_ = 4.0 nm (3.4 nm) and *g* = 30.2% (33.1%) for the (100) peak in the low-*M*_w_ (high-*M*_w_) batch, while for the (010) peak the numbers are 1.3 nm (1.4 nm) and 21.5% (21.0%) for the same batch. To quantify the amount of crystalline material in each case, we have performed an azimuthal integration of the (010) reflection while applying the Lorentz factor^40,41^ sin(*χ*) to the scattered intensity (Fig. S13c and f); that same calculation was also performed for the (100) reflection (see Table S3 and Fig. S14g). The corresponding Hermans orientation parameters obtained in the neat PTB7-Th films (and also in blends with IEICO-4F, *S*_100_ and *S*_010_; Table S3, Fig. S14c and f) corroborate the face-on texture of the films in all scenarios. Furthermore, our results indicate that in neat PTB7-Th films, a higher *M*_w_ yields a comparatively larger number of crystallites (quantified with *A*_100_ and *A*_010_ as the areas under the azimuthally-integrated linecuts). The integrated areas are 4.1 and 3.3 for *A*_010_ in the high- and low-*M*_w_ batches, respectively (Fig. S13c and f). This observation also holds true for the (100) reflections (*A*_100_; see Table S3 and Fig. S14g). These crystals are, however, of poor quality and coherence (*g* > 20% for (010), *g* > 30% for (100)). On the other hand, increasing the *M*_w_ preserves the quality of the crystals (comparable CCLs and *g* values). Therefore, any improvement in the charge carrier mobility observed for neat PTB7-Th films as a function of *M*_w_ can be at least partly supported by the existence of a comparatively larger fraction of (π-stacked) crystals in the film with the higher *M*_w_, and we can safely ignore any improvement derived from the quality of the crystals.

**Fig. 3:**
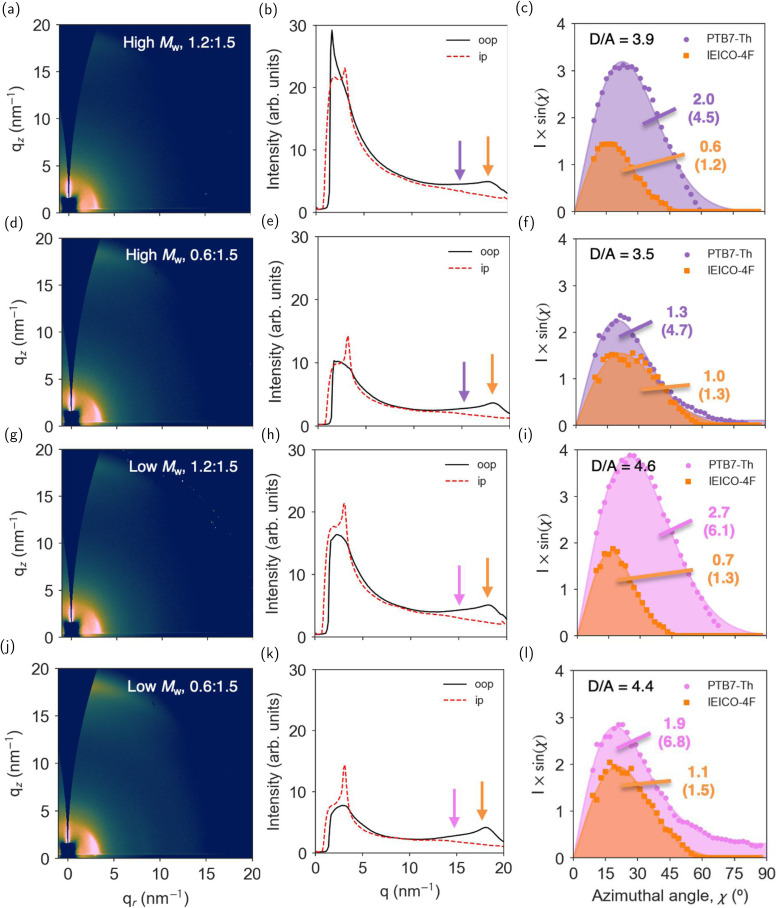
(a), (d), (g) and (j) 2D GIWAXS images of PTB7-Th : IEICO-4F blend films of distinct molecular weight and D : A ratio, as deposited on silicon. (b), (e), (h) and (k) The corresponding out-of-plane and in-plane linecuts, with the arrows indicating the positions of the (010) reflection in PTB7-Th (purple, violet) and IEICO-4F (orange). (c), (f), (i) and (l) Azimuthal analysis of the scattering intensity of the (010) reflections of both materials, with the resulting area under the curve also indicated. The number in brackets corresponds to the area obtained after correction for the actual mass of scatterer in the volume probed by the X-rays. D/A corresponds to the ratio between the corrected areas of the donor and acceptor.

Fig. 3a, d, g and j show the 2D GIWAXS images of PTB7-Th : IEICO-4F blends with different molecular weights and D/A composition ratios. It is first observed that the face-on texture found originally in the neat PTB7-Th films is preserved (according to the *S*_100_ and *S*_010_ values; Fig. S14c and f) yet an additional reflection appears primarily in the out-of-plane direction (Fig. 3b, e, h and k), here assigned to the π–π stacking of (010) planes of IEICO-4F and indicated with an orange arrow. In blends with a D/A ratio of 1.2 : 1.5, said reflection is centered at *q* = 18.3 ± 0.5 nm^−1^, while in blends with a D/A ratio of 0.6 : 1.5, the peak is shifted to *q* = 17.7 ± 0.6 nm^−1^, which indicates that the packing of IEICO-4F is affected by the polymer loading. In the low *q* range and in-plane direction, the characteristic lamellar reflection of IEICO-4F at *q* = 3.2 ± 0.1 nm^−1^ co-exists with the (100) reflection of PTB7-Th at *q* = 2.7 ± 0.1 nm^−1^. Following the same rationale as in neat PTB7-Th films, we have performed a careful quantitative analysis of the scattered intensity of the (100) and (010) reflections in the blends. Here, however, we have deconvoluted the azimuthal linecuts according to the material-specific reflections assigned to PTB7-Th and IEICO-4F, resulting in two distinct distributions of crystallites in the thin films; Fig. 3c, f, i and l illustrate such distributions for the (010) reflections. In each case, we have calculated the area under the curves and applied a normalization by the actual mass of scatterer found in the film volume probed by the X-rays, with the resulting (normalized) value indicated in brackets in Fig. 3c, f, i and l. This normalization aims at considering the actual X-ray scattering footprint across films of different D/A composition ratios, thus serving as a relative degree of crystallinity per unit mass of scatterer. This same process was also performed for the characteristic (100) reflections (Table S3 and Fig. S14g).

From the quantitative analysis of the (100) peaks (Fig. S14a–c), both the *L*_c_ and the *g* of PTB7-Th point to a massive enhancement of the crystal quality along the lamellar stacking direction upon blending with IEICO-4F: the *L*_c_ increases to 14–19 nm (*cf.* 3–4 nm in neat PTB7-Th films) while *g* decreases to 14.7–16.5% (*cf.* 30.2–33.1% in neat PTB7-Th). Here, a higher *M*_w_ systematically yields larger *L*_c_ and lower *g* values, indicating improved quality of ordering in the PTB7-Th lamellae. On the other hand, the quantitative analysis of the (010) peak reveals further worsening of the crystal quality along the π-stacking direction regardless of the *M*_w_, with the *L*_c_ and *g* values being downgraded to 1.0–1.1 nm and 23–25%, respectively (*cf.* 1.3–1.4 nm and 21.0–21.5%).

Overall, the effect of the D/A ratio and the *M*_w_ of PTB7-Th on the quality of IEICO-4F crystals is minor, since the *L*_c_ and *g* values are similar across the different sample types (Table S3 and Fig. S14a–f). The (100) IEICO-4F reflections indicate *L*_c_ and *g* values of 9.2–12.4 nm and 16.1–18.8%, respectively, but a significantly enhanced (compared to that of PTB7-Th) *L*_c_ for the (010) reflections (2.6–3.0 nm) with *g* approaching 13.6–14.4%.

Quantitatively, PTB7-Th experiences a significant increase in the lamellar (100) reflection when blended with IEICO-4F, compared to the pristine film (Fig. S14g). Said areas (*A*_100_) are 12.7 (0.6 : 1.5) and 8.1 (1.2 : 1.5) for the high-*M*_w_ PTB7-Th blend, compared to the value of 4.0 obtained for the pristine film. For low-*M*_w_ PTB7-Th, those same areas are 7.1 (0.6 : 1.5) and 14.1 (1.2 : 1.5) *vs.* 3.5 in the pristine film. Thus, upon blending with IEICO-4F, a comparatively larger fraction of PTB7-Th crystallizes in lamellae. For the (010) reflections, the high-*M*_w_ blends show integrated *A*_010_ values of 4.7 (0.6 : 1.5) and 4.5 (1.2 : 1.5) *vs.* 4.1 in the pristine film; while low-*M*_w_ blends have values of 6.8 (0.6 : 1.5) and 6.1 (1.2 : 1.5) *vs.* 3.3 in the pristine films. In the case of IEICO-4F, the *A*_010_ values are 1.3 (1.5) and 1.2 (1.3) in the same high-*M*_w_ (low-*M*_w_) scenarios. This implies that (i) blending with IEICO-4F increases the fraction of (100) and (010) diffracting planes attributed to PTB7-Th; (ii) the use of lower-*M*_w_ PTB7-Th in the blend (regardless of the D/A ratio) nearly doubles the amount of (010) planes compared to the pristine film, while higher-*M*_w_ PTB7-Th constrains such an increase to *ca.* 10% only; and (iii) the crystallinity of IEICO-4F is determined by a combination of its mass loading in the blend (the higher the loading, the more IEICO-4F crystallites) and the *M*_w_ of PTB7-Th (the lower the *M*_w_, the more IEICO-4F crystallites).

Our GIWAXS analysis suggests that the choice of D/A ratio has limited influence on the *A*_010_ parameters of PTB7-Th in the blend (Fig. S14h). We thus consider that the key differences, if any, in the structure–property dyad of PTB7-Th could be attributed to those observed in *A*_100_. The best charge carrier mobility for holes and electrons is observed for the 0.6 : 1.5 blend using high-*M*_w_ PTB7-Th, which according to our GIWAXS analysis correlates with a high *A*_100_ (12.7). For that same D/A ratio, the use of lower-*M*_w_ PTB7-Th nearly halves *A*_100_ (7.1). This suggests that a larger proportion of PTB7-Th (100) planes could benefit the charge transport. Notwithstanding, as the polymer loading increases to 1.2 : 1.5 (D/A), the *A*_100_ area is reduced to 8.1 in the high-*M*_w_ case. In this case, using PTB7-Th of low-*M*_w_ increases *A*_100_ to a record 14.1. Still, the charge carrier mobility observed in this scenario shows the worst values (Table S3), which would support the idea that *A*_100_ (when referenced to PTB7-Th) is not a suitable parameter to properly describe the structure–property relationship in PTB7-Th : IEICO-4F blends. Instead, the properties of IEICO-4F could explain the observed charge carrier mobility trend (*vide infra*).

We have calculated the *A*_010_ ratio between the donor and acceptor scattering signals, as indicated in Fig. 3c, f, i and l in the top-left corners, introduced here as a metric to quantify if the relative degree of crystallinity between donor and acceptor materials varies across films. The D/A scattering ratios are systematically higher in low-*M*_w_ (4.4–4.6) than in high-*M*_w_ PTB7-Th : IEICO-4F blend films (3.5–3.9). With the noted increase in the hole mobility when using high-*M*_w_ PTB7-Th and the comparatively higher crystallinity (for both donor and acceptor, see Fig. S14g and h) attained in the blend films containing low-*M*_w_ PTB7-Th, it thus becomes clear that over large distances (150–300 nm, as employed in SCLC devices), the actual *M*_w_ of PTB7-Th is the key factor that enables improved charge transport and not its relative degree of crystallinity. Moreover, there exists a positive correlation between a larger mass fraction of IEICO-4F in the blend (0.6 : 1.5) and enhanced electron and hole mobilities in all cases, which according to our quantitative GIWAXS analysis correlates also with a larger fraction of IEICO-4F crystals in the blend films. Thus, the optimum charge carrier mobility in SCLC devices (namely, with high-*M*_w_ PTB7-Th and a D/A ratio of 0.6 : 1.5) synergistically benefits from (i) the improved percolation due to the high *M*_w_ of PTB7-Th, and (ii) the enhanced IEICO-4F crystallinity as its ratio increases in the blend.

#### 2.2.6 Transparency

**Fig. 4:**
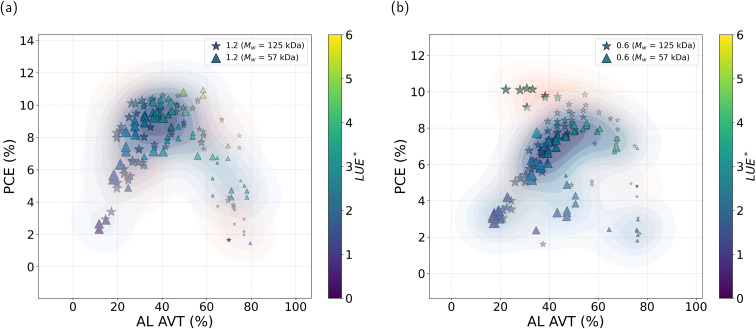
PCE and AL light utilization efficiency (LUE*) as a function of AL AVT for devices with PTB7-Th/IEICO-4F ratios at (a) 1.2 : 1.5 and (b) 0.6 : 1.5 and different molecular weights (57 kDa and 125 kDa).

In this section we discuss the connection between AL transparency and device performance. Previous studies, such as those by Zhou *et al.*^36^ and Meng *et al.*,^24^ investigated the relationship between D/A ratio, solute concentration, and AVT in PTB7-Th and IEICO-4F, while Yue Zang *et al.*^23^ emphasized enhanced light utilization in devices based on IEICO-4F. Together, these studies underscore the effectiveness of this material system for high-performance semitransparent solar cells. The AVT of the device can be primarily influenced by the donor/acceptor (D/A) ratio and film thickness. The interplay between transparency and device efficiency in our study is illustrated in Fig. 4. This analysis includes all opaque devices fabricated during this investigation, covering both 0.6 : 1.5 and 1.2 : 1.5 donor/acceptor ratios, as well as 57 kDa and 125 kDa molecular weights of PTB7-Th. The two D/A ratios show distinct performance characteristics considering AL AVT, PCE and AL light utilization efficiency (LUE*). LUE* denotes the light utilization efficiency of the device, accounting solely for the contributions of the glass substrate, ITO, electron transport layer (ETL), and AL. It is calculated based on the light transmitted through these layers and the corresponding PCE for opaque solar-cell devices.

In this section, LUE* is defined for the glass/ITO/ETL/AL only to enable systematic comparison between donor/acceptor ratios. The full-stack transparency and LUE, including top electrodes, are reported separately in Section 2.3.4 and Table 4.

For the 1.2 : 1.5 ratio, the PCE values are similar for both molecular weights (57 and 125 kDa), with slightly better performance for devices with higher *M*_w_ at low and high AL AVT (thicker and thinner, respectively). A small difference to notice is the fact that devices with a molecular weight of 125 kDa show greater device processability, as evidenced by more devices that function at AVT values above 60%.

In contrast, for the 0.6 : 1.5 ratio, reducing the PTB7-Th content results in a clear advantage for the higher-molecular-weight blend, which maintains a comparable PCE to that of the 1.2 : 1.5 ratio. This improvement is particularly pronounced at higher AVT values. Notice, for instance, that the upper crust of the data clouds is dominated by star symbols (Fig. 4b). We can also observe the trends in FF and *J*_sc_ in Fig. S15. For the 1.2 : 1.5 ratio, the decrease in PCE at low AVT is primarily related to a decrease in FF, while the decrease at high AVT is driven by lower *J*_sc_. For the 0.6 : 1.5 ratio, higher-molecular-weight devices exhibit better *J*_sc_ values, allowing a reduced PTB7-Th content without a significant loss of photocurrent, a key observation indicating the benefits of higher molecular weight in improving charge transport and light absorption. LUE* provides an additional context to assess the interaction between transparency and PCE. For the 1.2 : 1.5 ratio, the LUE* remains robust near 60% AVT, supported by the high PCE across both molecular weights. For the 0.6 : 1.5 ratio, the overall LUE* is slightly lower than for the 1.2 : 1.5 ratio, probably due to the significant influence of the PCE on the LUE* calculation. However, the higher-molecular-weight devices that exceed the 60–80% AVT range achieve a higher concentration of points above 6% PCE. This suggests that the combination of reduced D/A ratio and increased molecular weight offers a viable route to highly transparent and efficient devices.

#### 2.2.7 Blending molecular weight batches

*M*_w_ blends may help to optimize the balance between charge transport and transparency. Low-molecular-weight polymers in some cases offer better transparency but may suffer from poor charge transport, while high-molecular-weight polymers improve charge transport but may reduce transparency. For instance, Nan Wei *et al.*^42^ demonstrated that blending high- and low-molecular-weight components can yield an ideal phase morphology. Specifically, they observed that by adding 10% low-molecular-weight D18 to high-molecular-weight D18, a multiscale interpenetrating fiber network structure within the active layer was successfully created, leading to significant improvements in device performance. To explore the potential advantages of combining low- and high-molecular-weight PTB7-Th, we fabricated devices with six different blends, ranging from 100% low molecular weight (L100_H0) to 100% high molecular weight (L0_H100). Two separate solutions of PTB7-Th and IEICO-4F were prepared, one with low molecular weight and the other with high molecular weight, and then mixed in various proportions to create intermediate blends.

Fig. S17 shows the PCE as a function of the average visible transmittance (AVT) for these blends, allowing us to evaluate their performance with varying transparency. For thicker ALs (lower AVTs), the PCE results are generally similar across all blends. However, the most notable differences appear in the devices with the thinnest AL. At 70% AL AVT, the devices fabricated with 100% high (L0_H100) and low (L100_H0) *M*_w_ achieve PCEs close to 4% and 2% respectively. Notably, the intermediate blends demonstrate better performance compared to both pure low- and high-*M*_w_ samples under these conditions. In particular, the L10_H90 and L30_H70 blends exhibit the highest efficiencies, reaching PCE values close to 8% at AL AVT levels around 70%. These results suggest that molecular weight blends may offer a better balance between morphology, charge transport, and transparency, especially for thin ALs. It is important to note that this was an isolated experiment designed to evaluate the potential of intermediate molecular-weight blends. While the results are promising, further studies are required to validate these findings and understand the underlying mechanisms contributing to their higher performance.

### 2.3 Processability and upscaling

An iterative study was conducted to evaluate the impact of critical fabrication parameters to assess the scalability of the optimized PTB7-Th : IEICO-4F blend (high molecular weight with a 0.6 : 1.5 D/A ratio). Each step was built upon previous findings, ensuring controlled progression toward practical device fabrication. The experiments aimed to balance high power conversion efficiency (PCE) with large-area scalability and long-term stability under realistic manufacturing conditions. A summary of the *J*–*V* characteristics of the champion devices manufactured under these varied conditions is provided in Table 3. In short, we tested processing in air, different solvents, use of additives, semitransparent electrodes, cell area, and module fabrication. We discuss the main findings in the following paragraphs and subsections.

**Table 3:** Photovoltaic parameters of champion solar cells fabricated under various conditions. Fabrication parameters include solvent type (CB: chlorobenzene, OX: *o*-xylene), donor/acceptor ratio, donor molecular weight, fabrication atmosphere (IN: nitrogen glovebox, OUT: ambient), additive content, and electrode type (OP: opaque, ST: semitransparent)

Solvent	Ratio	*M*_w_ (kDa)	Atm	Add (%)	Electrode	*V*_oc_ (V)	*J*_sc_ (mA cm^−2^)	FF (%)	PCE (%)	*R*_s_ (Ω)	*R*_sh_ (Ω)
CB	1.2	125.2	IN	0	OP	0.72	21.3	58.4	8.9	62.9	3791
CB	1.2	125.2	IN	4	OP	0.69	23.5	64.0	10.3	45.4	6295
CB	1.2	57.5	IN	4	OP	0.69	23.3	67.6	10.9	42.0	7254
CB	1.2	125.2	IN	0	ST	0.72	19.9	55.9	8.0	82.8	5804
CB	1.2	125.2	IN	1	ST	0.70	19.2	63.8	8.5	67.4	6232
CB	1.2	125.2	IN	4	ST	0.67	18.4	60.2	7.5	63.1	4789
CB	0.6	125.2	IN	0	OP	0.69	20.9	65.7	9.5	52.0	5548
CB	0.6	125.2	IN	4	OP	0.69	21.5	68.1	10.2	41.3	4809
CB	0.6	57.5	IN	4	OP	0.68	18.5	66.2	8.3	47.5	10 492
CB	0.6	125.2	OUT	0	OP	0.72	21.9	62.5	9.8	46.4	4242
CB	0.6	125.2	IN	0	ST	0.71	19.6	53.4	7.4	89.2	4778
CB	0.6	125.2	IN	1	ST	0.69	18.5	63.8	8.1	57.0	4941
CB	0.6	125.2	IN	4	ST	0.67	19.5	39.7	5.2	254.4	1595
OX	1.2	125.205	OUT	0	ST	0.71	18.6	58.6	7.7	73.6	5924
OX	1.2	57.467	OUT	0	ST	0.69	19.4	61.2	8.3	64.4	4735
OX	0.6	125.205	OUT	0	ST	0.68	19.6	61.3	8.2	65.1	6418
OX	0.6	57.467	OUT	0	ST	0.67	18.9	62.6	7.9	62.8	6688
OX	0.6	57.467	OUT	0	OP	0.69	23.2	52.7	8.4	66.0	3458
OX	0.6	125.205	OUT	0	OP	0.69	23.7	59.8	9.8	48.9	3769
OX	0.6	125.205	IN	0	OP	0.71	22.5	58.3	9.3	49.0	3861

Table S1 shows the percentage of successfully fabricated devices for each combination of D/A ratio and molecular weight (*M*_w_), highlighting the processability differences between high- and low-molecular-weight samples. These results further illustrate the advantages of high-*M*_w_ PTB7-Th. High-*M*_w_ devices consistently exhibit higher fabrication yields across all donor ratios, with particularly notable differences at 0.4 : 1.5, 0.6 : 1.5 and 1.5 : 1.5. The yield for high-*M*_w_ devices reaches 63%, 81% and 92%, respectively, compared to 21%, 60% and 25% for low *M*_w_. This enhanced processability underscores the scalability potential of high-*M*_w_ PTB7-Th for large-scale production.

#### 2.3.1 Environmental conditions

The next parameter examined was the manufacturing environment. The devices from previous sections were initially fabricated inside a nitrogen filled glovebox. Now, the fabrication was carried out under ambient air conditions. This transition is a critical milestone for scalability, enabling production under more accessible and cost-effective conditions. Maintaining device performance under ambient conditions reflects the resilience of the material system and its suitability for industrial production.^43^ As illustrated in Table 3, the devices manufactured in this way exhibit a small efficiency loss. For instance, a PCE of about 9.8% compared to 10.2% is obtained for the 0.6 : 1.5 D/A ratio. It should be noted that other key performance metrics, including *J*_sc_ and *V*_oc_, exhibited minimal changes between the two sets of conditions. These results show the robustness of the selected PTB7-Th : IEICO-4F blend to exposure to oxygen and moisture during the fabrication process.

#### 2.3.2 Solvent

Once the environmental conditions were fixed outside the glovebox, the next step was to replace chlorobenzene (CB) with *o*-xylene (OX), a more environmentally friendly solvent.^44^ High-molecular-weight devices fabricated inside and outside the glovebox with OX achieved a PCE of 9.3% and 9.8%, respectively, compared to 10.2% and 9.8% with chlorobenzene (CB). So, switching from CB to OX resulted in minor performance trade-offs for high-molecular-weight devices and provided comparable efficiencies under more scalable and environmentally friendly conditions in terms of global warming potential (GWP).^44^ Proving the processability of any photoactive materials system using industrially compatible solvents is of high importance for large-scale roll-to-roll processing.

#### 2.3.3 Additive content

In order to optimize device performance, and following the literature,^45^ chloronaphthalene (CN) was added to the blends at different concentrations. The devices were fabricated using PTB7-Th of 125 kDa and 57 kDa *M*_w_ with 0.6 : 1.5 and 1.2 : 1.5 D/A ratios. The PV parameters are summarized in Table 3. For the 1.2 : 1.5 D/A ratio, the addition of 4% CN significantly improved the photocurrent (from around 21 mA cm^−2^ to 23 mA cm^−2^) and FF (from 58% to 64%), resulting in higher PCE values. This improvement was consistently observed in both higher- and lower-molecular-weight-based devices, indicating that the additive enhances charge generation. However, white stains (indicative of increased roughness) were observed on the electrodes of the samples with 4% CN (Fig. S18). These morphological changes were relatively minor for devices with the 1.2 : 1.5 D/A ratio and did not hinder device reproducibility. In contrast, for the 0.6 : 1.5 D/A ratio, where the donor content is reduced in the blend, the impact of the additive was more pronounced. Devices with 4% CN exhibited reduced reproducibility and processability, with a larger variance in performance metrics. The presence of white stains was more pronounced in devices of high molecular weight with 4% CN and even more in devices based on lower *M*_w_. This observation indicates that the additive exhibits a stronger interaction with the acceptor (IEICO-4F) at reduced donor concentrations, resulting in stronger phase separation and compromising device performance.

To further investigate the nature of the white stains, Raman spectroscopy was performed on the affected areas. Microscopy images of the samples with the 0.6 : 1.5 D/A ratio (Fig. S19) show that the high-molecular-weight donor results in a more homogeneous active layer, while the low-molecular-weight donor exhibits segregation and inhomogeneity. Raman spectra (SI) comparing normal active-layer regions and regions with white stains reveal that, for high-molecular-weight donors, the Raman signals are very similar. However, for low-molecular-weight donors, there are noticeable differences in the peaks, with the PTB7-Th peaks being more prominent in the white-stain regions compared to the reference peaks of the materials (Fig. S20 and S21). These results suggest that the white stains correspond to an accumulation of the donor in these areas.

In conclusion, while the use of additives such as CN can enhance photocurrent and efficiency for the 1.2 : 1.5 ratio by promoting better charge transport, their effect on morphology becomes detrimental for low donor content (0.6 : 1.5), particularly in blends with low-molecular-weight PTB7-Th. Importantly, high *M*_w_ enables higher PCEs for low donor content without additive, which greatly favors scalability and stability.

#### 2.3.4 Semitransparent electrodes

The integration of semitransparent electrodes represents a critical step in the development of devices suitable for applications such as BIPVs and agrivoltaics. The semitransparent solar cells used in this study employed the following electrode configuration: glass/ITO as the substrate and bottom electrode, 30 nm ZnO as the ETL, a gradient AL, 15 nm MoO_*x*_ as the HTL, and 20 nm Ag as the semitransparent top electrode. As shown in Table 3, the transition from opaque to semitransparent devices results in a decrease in performance metrics. The *J*_sc_ is reduced from approximately 23 mA cm^−2^ to 18 mA cm^−2^, while the FF drops from around 63% to 60%. Consequently, the maximum PCE achieved decreases from 10% to 8%. To further analyze the impact of semitransparent electrodes, Table 4 summarizes the PV parameters (including *V*_oc_, *J*_sc_, FF, PCE, AVT, and Light Utilization Efficiency (LUE)) for champion devices fabricated with semitransparent electrodes (AVT and LUE values in the table correspond to the complete semitransparent stack). These include devices with D/A ratios of 1.2 : 1.5 and 0.6 : 1.5 and low- and high-molecular-weight PTB7-Th. Interestingly, the reduction in *J*_sc_ from the opaque to semitransparent devices is consistent across both 1.2 : 1.5 and 0.6 : 1.5 ratios. Consequently, under these conditions, we can achieve equivalent PCEs with both ratios. Despite the reduction in PCE for semitransparent devices, the results for AVT and LUE reveal the advantage of reducing the donor content and increasing the molecular weight. Among the tested configurations, the 0.6 : 1.5 ratio with high-molecular-weight PTB7-Th achieves the best overall performance in terms of transparency and LUE. This is further illustrated in Fig. S22, which presents the *J*–*V* curves of the champion semitransparent devices, highlighting their performance under standard illumination conditions. We benchmarked the performance of our best semitransparent device against representative PTB7-Th : IEICO-4F-based semitransparent OPVs reported in the literature. A comparison of PCE, AVT and full-stack LUE is provided in Table S5 (SI). The maximum LUE obtained in our devices (2.1%) is lower than those reported for some fully optimised spin-coated semitransparent OPVs. This is expected because, in this study, no optimisation of the semitransparent electrode was performed and our devices were fabricated by blade coating rather than spin coating. The Ag electrode used in our case is slightly thicker than in typical ST-OPV designs, resulting in reduced visible transmittance. We note that the primary objective of this work was to investigate the transparency and optoelectronic response of the active layer, rather than to optimise the optical performance of the transparent electrode. As absorption is cumulative, we expect that the comparative gains in active-layer AVT and LUE* will translate into better-performing semitransparent cells in other device geometries too, such as when using more transparent electrodes. The improved thickness resilience may also be used to compensate for the lack of back reflection from more transparent back electrodes.

**Table 4:** PV parameters of the champion solar cells with varying donor molecular weight and ratio for semitransparent solar cells. In this table, AVT and LUE values were measured for the complete semitransparent stack

Ratio D/A	*M*_w_ (kDa)	*V*_oc_ [V]	*J*_sc_ [mA cm^−2^]	FF [%]	PCE [%]	*R*_s_ [Ω]	*R*_sh_ [Ω]	AVT [%]	LUE [%]
1.2 : 1.5	125.205	0.71	18.6	58.6	7.7	73.6	5924	23.9	1.8
1.2 : 1.5	57.467	0.69	19.4	61.2	8.3	64.4	4735	22.6	1.9
0.6 : 1.5	125.205	0.68	19.6	61.3	8.2	65.1	6418	25.8	2.1
0.6 : 1.5	57.467	0.67	18.9	62.6	7.9	62.8	6688	21.7	1.7

In summary, the implementation of semitransparent electrodes results in a reduction in certain performance metrics compared to those of opaque devices. However, optimization of the D/A ratio and molecular weight serves to partially mitigate these losses and enhance transparency.

#### 2.3.5 Single cell area

**Fig. 5:**
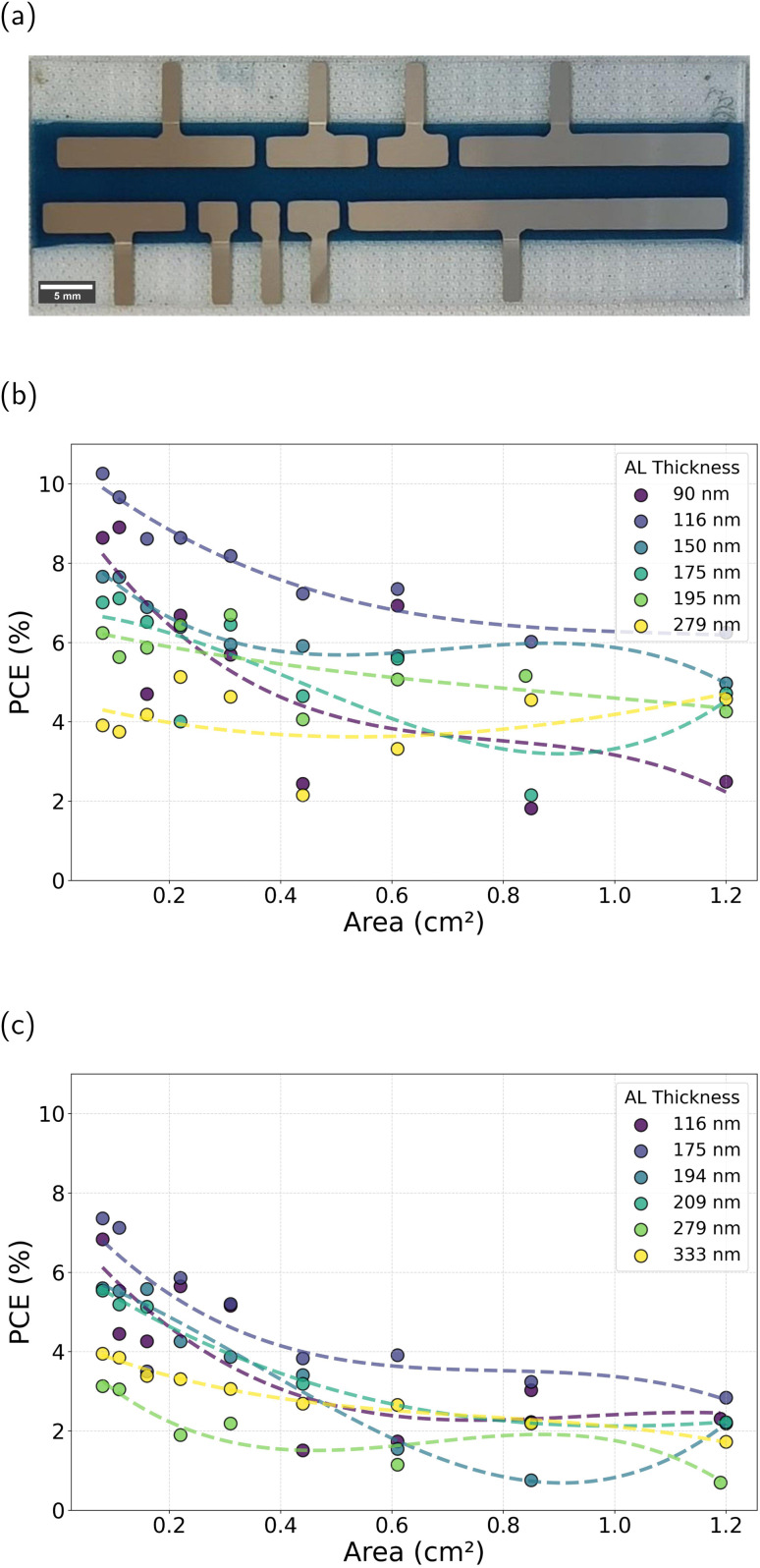
(a) Diagram of the samples with different areas. (b) and (c) Power conversion efficiency (PCE) of devices as a function of device area for (b) opaque and (c) semitransparent configurations with different AL thicknesses.

Scaling up device area is a crucial step for large-scale applications. To study the impact of device area, opaque and semitransparent devices, with AL thicknesses ranging from 90 to 279 nm for opaque devices and from 116 to 333 nm for semitransparent devices, were fabricated. For each thickness, devices with areas ranging from 0.08 cm^2^ (reference size) to 1.2 cm^2^ were fabricated on the same substrate, as illustrated in Fig. 5a. The relationship between PCE and device area for opaque and semitransparent configurations is presented in Fig. 5b and c, respectively. In agreement with previous sections in which each substrate exhibited a thickness gradient, the PCE for 0.08 cm^2^ (reference size) devices was 10% for opaque devices and close to 8% for semitransparent devices. For opaque devices, the optimum AL thickness was 116 nm, while for semitransparent devices, the optimum thickness was 175 nm. This difference is attributed to the role of the top electrode's reflectance, which doubles the optical path in opaque devices. Semitransparent devices require a thicker active layer to achieve comparable photocurrent values due to the lower light reflection at the back electrode.

In both configurations, a consistent trend is observed: PCE decreases as the device area increases, with a sharper decline for smaller areas (below 0.4 cm^2^). Beyond a certain point, the reduction stabilizes, indicating that certain limitations such as charge collection and uniformity are mitigated for larger areas. This suggests that current constraints imposed by the thin electrodes in semitransparent configurations outweigh the effects of AL thickness, leading to a reduced dependence on the thickness for larger areas. Additional insights into the impact of device area on other PV parameters, including *V*_oc_, *J*_sc_, FF are provided in Fig. S23 (opaque) and S24 (ST). A noticeable reduction in *J*_sc_ and FF with increasing device area is observed, consistent with findings reported by Agrawal *et al.* in their study on the efficient up-scaling of organic solar cells.^46^ These figures highlight the dependence of photovoltaic performance metrics on the scale and transparency characteristics of the device.

**Fig. 6:**
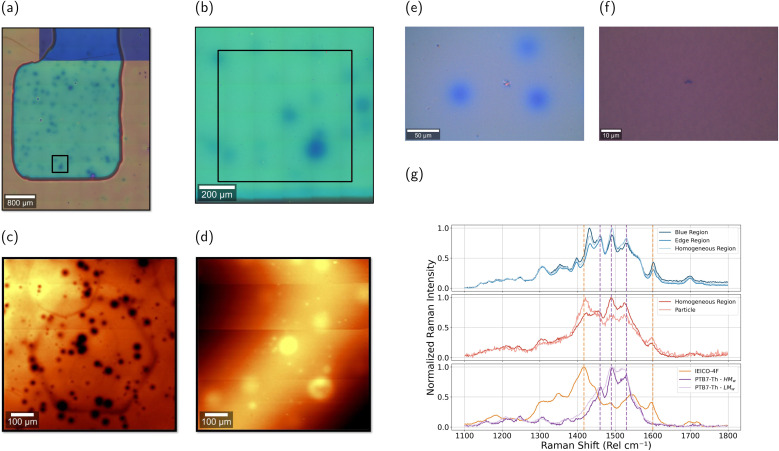
(a) Microscopy at 10× and (b) 40×, (c) LBIC and (d) PL images of an 8 mm^2^ pixel. (e) Microscopy image (40×) showing blue regions in the active layer, (f) microscopy image (100×) highlighting material particles embedded in the active layer and (g) Raman spectrum comparison between the blue regions and particles with the homogeneous active layer for a 0.6 : 1.5 D/A ratio sample, with the reference Raman spectra of the materials.

To further investigate the efficiency loss observed in large-area devices, microscopy, light-beam-induced current (LBIC) and photoluminescence (PL) imaging measurements were conducted on 8 mm^2^ and 60 mm^2^ pixels. Fig. 6a–d present microscopy images, LBIC, and PL maps for an 8 mm^2^ device, while data for the 60 mm^2^ device is provided in the SI (Fig. S27).

In this series of imaging data, two types of defects were identified: blue regions, more prominently in the large-area device, and solid material particles, detected in high-magnification images (40×). LBIC mapping revealed that these small material particles are directly correlated with localized photocurrent loss, acting as recombination centers and impeding charge collection. This effect was consistently observed in both 8 mm^2^ and 60 mm^2^ devices. PL measurements further confirmed the impact of these defects. Although material particles showed minimal PL variation, the blue regions in large-area devices (S27) exhibited higher PL intensity, suggesting morphological inhomogeneities.

To understand the nature of the defects identified in the large-area devices, Raman spectroscopy was performed on both the material particles and the blue regions. Fig. 6e–g present microscopy images along with the corresponding Raman spectra for each type of defect and the reference materials. In particular, for the blue-colored regions (Fig. 6e), three distinct regions were analyzed: the homogeneous (defect-free) region, the edge of the blue spot, and the center of the blue spot. When comparing the Raman spectrum from the blue spot with that of the homogeneous region, the peaks associated with IEICO-4F are enhanced, suggesting that the blue region is slightly richer in the acceptor. In contrast, at the edges of the blue spot, the IEICO-4F peaks are slightly reduced relative to the homogeneous region, which implies that there is less acceptor present at the edges of the spot. This observation suggests that IEICO-4F may diffuse into the blue region, thereby altering the local composition and contributing to the formation of these defects.

For the material particles (Fig. 6f), the Raman spectrum revealed an enhanced signal in the vibrational modes associated with the acceptor (IEICO-4F) when compared to the reference blended active layer. This suggests that these particles are acceptor-rich aggregates. Such compositional inhomogeneities can disrupt percolation pathways, impeding charge extraction, and increasing recombination, as previously observed in LBIC measurements.

To quantitatively correlate defect formation with performance, microscopy images (10×) were acquired from four pixels (two thick and two thin) for each sample under various conditions (different D/A ratios, molecular weights, and CN additive contents). The defect density (defects per mm^2^) was calculated, and the results are presented in Table S6. For the 0.6 : 1.5 ratio, the lowest defect density was observed in high-molecular-weight samples without CN additive (12 def. per mm^2^). In contrast, the addition of 4% CN increased defect density for both high- and low-molecular-weight samples. For the 1.2 : 1.5 D/A ratio, thicker layers exhibited a significantly higher defect density. However, in thinner layers with 4% CN additive, defect density was lower than that observed in the 0.6 : 1.5 samples, particularly for high-molecular-weight formulations. This suggests that the CN additive effectively enhances film uniformity at this D/A ratio, especially in thinner layers.

Additionally, thicker ALs consistently showed a higher defect density, reinforcing the idea that increased thickness leads to more inhomogeneous film formation and particle aggregation. This trend is further supported by the defect size distribution histograms in the SI (Fig. S34 and S36), which show the frequency of defect diameters for different conditions. The histograms reveal that for thicker layers, not only is the total defect density higher (as observed in Table S6), but there is also a greater proportion of larger particles. In contrast, thinner ALs exhibit predominantly small defects, particularly in the 0.6 : 1.5 high-molecular-weight sample without CN additive, where nearly 100% of defects measure below 5 µm. These findings suggest that film formation in thinner layers is more uniform, whereas increased thickness promotes larger aggregation sites. This correlation aligns with the processability trends observed in Table S4, where low-molecular-weight 0.6 : 1.5 samples exhibited poorer processability compared to high-molecular-weight devices. Further studies on the nature of defect formation as well as mitigation strategies are needed.

#### 2.3.6 Modules

**Fig. 7:**
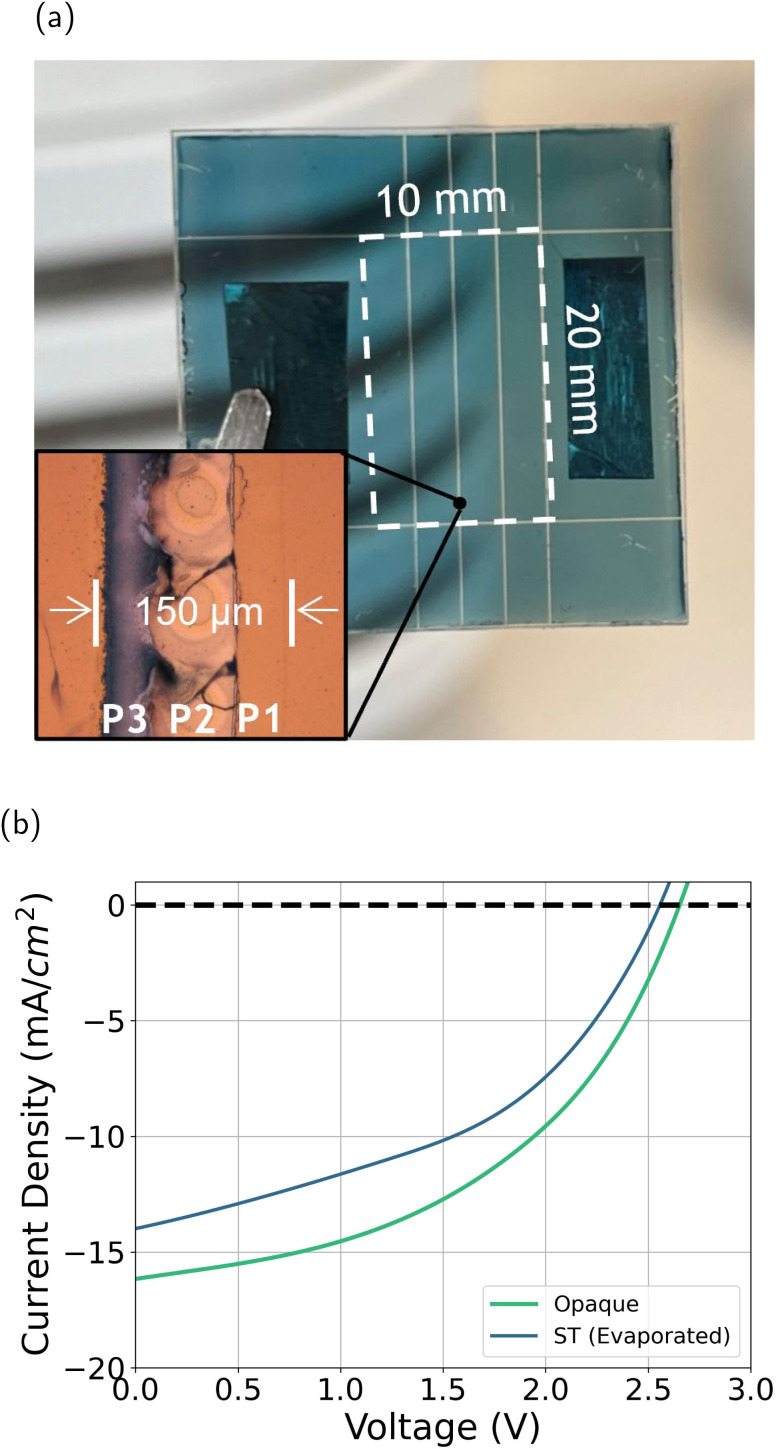
Overview of module fabrication and performance. (a) Photograph of a semitransparent module, with a close-up view of the laser-patterned interconnections (P1, P2, P3); the active area of the module is marked in white dashed lines. (b) *J*–*V* curves for the fabricated modules (opaque and ST with evaporated electrodes).

In order to assess the scalability and performance of the optimized PTB7-Th : IEICO-4F blend in larger-scale devices, two modules were fabricated: one opaque module with 10 nm MoO_*x*_ and 100 nm Ag and one semitransparent module with an evaporated HTL and electrodes (10 nm MoO_*x*_ and 20 nm Ag). Each module was composed of four subcells, with total areas of 2 cm^2^ for the opaque module and 2 cm^2^ for the evaporated semitransparent module. All modules were fabricated using the same active layer (AL) and fabrication conditions as in previous sections for semitransparent devices. Fig. 7a shows a photo of the semitransparent module with a zoomed-in view of the interconnections. In Fig. S37, a schematic of the laser-based interconnections is shown, where P1 separates the ITO for the different subcells, P2 breaks the intermediate layers to allow the top electrode to connect with the bottom electrode, and P3 separates the top electrode for the different subcells. Finally, Fig. 7b presents the *J*–*V* curves of the fabricated modules.

The performance of the modules, as summarized in Table 5, shows a clear trend similar to that observed in the semitransparent-electrode section. The opaque module achieved the highest PCE of 5.2%, while the semitransparent module with evaporated electrodes showed a reduced PCE of 4.0%. This reduction in efficiency is primarily due to a decrease in *J*_sc_ and FF.

**Table 5:** PV parameters of fabricated modules with different configurations and the largest-area devices of the device-area section. The table includes the number of subcells, total active area, geometric fill factor (GFF), *V*_oc_, *V*_oc_ per cell, *J*_sc_, FF, and PCE

Split cell/module	Top electrode	No. subcells	Area (cm^2^)	GFF	*V*_oc_ (V)	*V*_oc_ per cell (V)	*J*_sc_ (mA cm^−2^)	*V*_mpp_ (V)	*I*_mpp_ (mA)	*P*_mpp_ (mW)	FF (%)	PCE (%)
Split cell	Opaque	—	1.2	—	0.70	0.70	18.6	—	—	—	46	6.0
Split cell	ST (evaporated)	—	1.2	—	0.69	0.69	14.0	—	—	—	34	3.2
Module	Opaque^a^	4	2.0	92	2.65	0.66	16.8	1.77	5.6	9.9	46	5.2
Module	ST (evaporated)^b^	4	2.0	94	2.56	0.64	14.5	1.75	4.5	7.8	43	4.0

a Ag: 100 nm.

b Ag: 20 nm.

To further contextualise the performance of the modules, Fig. S25 compares the PCE of the module and of the single pixels as a function of device area. For both opaque (Fig. S25a) and semitransparent (Fig. S25b) architectures, the small-area devices exhibit the highest efficiencies, which gradually decrease as the active area increases. The module results have been superimposed onto these trends by marking the subcell area (0.5 cm^2^, filled star) and the total module area (2 cm^2^, hollow star). For the opaque architecture, both module points lie close to the extrapolated trend from the smaller-area devices, considering minor variations in active-layer thickness and additional losses introduced by the laser interconnections. This confirms that the reduced PCE of the opaque module is consistent with the intrinsic area dependence of the system. Interestingly, for the semitransparent architecture, the module performs slightly above the expected trend based on the area scaling measurements. This behaviour may arise from the lower photocurrents in semitransparent devices, which reduce resistive and interconnection losses compared to opaque devices. Overall, the module data follow the general scaling behaviour observed in the single cell area study, reinforcing that the differences in performance between opaque and semitransparent modules depend on area effects.

## 3 Conclusion

In this study, we systematically investigated the impact of D/A ratio, thickness and polymer molecular weight on the performance and transparency of organic solar cells. For low-*M*_w_-based devices, the 1.2 : 1.5 D/A ratio offered the highest overall efficiency but limited transparency, while reducing the donor content to 0.6 : 1.5 enhanced transparency but led to performance loss. By increasing the molecular weight of PTB7-Th from 57 to 125 kDa, the device performance was maintained even at low donor content. This improvement was linked to enhanced charge carrier mobility, as confirmed by SCLC measurements. The introduction of high-*M*_w_ PTB7-Th improved percolation, while reducing the polymer content improved acceptor crystallinity. In terms of transparency, higher molecular weight allowed devices to remain efficient at high AVT values, particularly for the 0.6 : 1.5 ratio. Importantly, we demonstrate the generality of the concept by investigating four other polymer/acceptor blends, showing that increasing *M*_w_ enables making efficient low-donor-content devices, and/or making them more thickness-resilient.

In terms of upscaling, *M*_w_ could not be associated with important changes in morphological stability, but reduced the number of defects on the films, which maintained most of the original efficiencies even when processed in air and from *o*-xylene. Increasing active-layer area and replacing the top electrode with a semitransparent one led to the most significant drops in PCE (from 10% to 5%).

## 4 Experimental section

### 4.1 Materials

The donor polymer used was PTB7-Th,††Poly[4,8-bis(5-(2-ethylhexyl)thiophen-2-yl)benzo[1,2-*b*;4,5-*b*′]dithiophene-2,6-diyl-*alt*-(4-(2-ethylhexyl)-3-fluorothieno[3,4-*b*]thiophene-)-2-carboxylate-2-6-diyl]. used in two molecular weights: low (*M*_w_ = 57.48 kDa, from 1-Material) and high (*M*_w_ = 125.21 kDa, from Ossila). The non-fullerene acceptor was IEICO-4F,‡‡2,2′-((2*Z*,2′*Z*)-(((4,4,9,9-Tetrakis(4-hexylphenyl)-4,9-dihydro-sindaceno[1,2-*b*:5,6-*b*′]dithiophene-2,7-diyl)bis(4-((2-ethylhexyl)oxy)thiophene-5,2-diyl))bis(methanylylidene))bis(5,6-difluoro-3-oxo-2,3-dihydro-1*H*-indene-2,1-diylidene))dimalononitrile. provided by Ossila. Zinc oxide (ZnO) ink (N-10) was purchased from Avantama. Molybdenum oxide (MoO_*x*_) was from Alfa Aesar. BM-HTL-1 was obtained from Brilliant Matters. Glass substrates with patterned indium tin oxide (ITO; 100 nm thick) were purchased from Ossila, enabling simultaneous fabrication of 24 devices per substrate. Chlorobenzene and *o*-xylene were used as solvents, and 1-chloronaphthalene (CN) as an additive. All were obtained from Sigma Aldrich and used without further purification. For the Generality of the concepts section, we used as donors the polymers PTQ10,§§Poly[[6,7-difluoro[(2-hexyldecyl)oxy]-5,8-quinoxalinediyl]-2,5-thiophenediyl]. and PM6.¶¶Poly[(2,6-(4,8-bis(5-(2-ethylhexyl)-4-fluorothiophen-2-yl)-benzo[1,2-*b*:4,5-*b*′]dithiophene))-*alt*-(5,5-(1′,3′-di-2-thienyl-5′,7′-bis(2-ethylhexyl)benzo[1′,2′-*c*:4′,5′-*c*′]dithiophene-4,8-dione))]. PTQ10 was used in three molecular weights: 63, 120 and 147 kDa, all of them from 1-Material. PM6 was used in two molecular weights: 83 kDa from Brilliant Matters and 106 kDa from Ossila. We also used as non-fullerene acceptors COTIC-4F, Y6 and DTY6, provided by 1-Material.

### 4.2 Device fabrication

Organic solar-cell devices were fabricated using an inverted structure of ITO/ZnO/AL/MoO_*x*_/Ag. The ITO glass substrates were subjected to a sequential cleaning procedure, sonicating in acetone (5 min), 2% Hellmanex solution in water (5 min), isopropanol (IPA) (5 min), and 10% sodium hydroxide (NaOH) solution (10 min), followed by rising in deionized (DI) water. For the ETL, zinc oxide (ZnO) was deposited using an automatic blade coater (Zehntner ZAA 2300) under ambient conditions, with a droplet volume of 50 µL, a blade gap of 50 µm and a constant speed of 5 mm s^−1^ at a controlled temperature of 40 °C. The ETL was then annealed at 120 °C for 10 minutes. The AL, consisting of a blend of PTB7-Th : IEICO-4F at a 20 mg mL^−1^ solution concentration, was blade coated inside of a nitrogen-filled glovebox to maintain controlled ambient air conditions or outside the glovebox for the upscalability experiments. The coating gap was set at 100 µm, volume at 60 µL and platform temperature at 80 °C. The blade speed was progressively decreased from 90 mm s^−1^ to 10 mm s^−1^ to achieve a thickness gradient across the substrate. Then, samples were annealed at 100 °C for 5 minutes inside of a nitrogen-filled glovebox or outside the glovebox for the upscalability experiments. A concentration of 20 mg mL^−1^ in CB or *o*-xylene (depending on the experiment) was used, with a donor/acceptor ratio ranging from 0.4 : 1.5 to 1.5 : 1.5. All samples were evaporated under ultra-high-vacuum conditions for the hole transport layer and top electrodes. Two different electrode configurations were used for opaque (OP) and semitransparent (ST) devices. OP samples with 5 nm MoO_*x*_ and 100 nm Ag layers were evaporated at a rate of 0.5 Å s^−1^ and 1.5 Å s^−1^, respectively. The electrode in ST devices consisted of 15 nm MoO_*x*_, 1 nm Au, and 20 nm Ag evaporated at a rate of 0.5 Å s^−1^, 0.1 Å s^−1^, and 0.5 Å s^−1^, respectively.

For the experiments included in Section 2.2.2, the devices were fabricated using the same processing conditions described above. All interlayers and the device architecture (ITO/ZnO/AL/MoO_*x*_/Ag) were kept identical. The active layers were prepared in chlorobenzene and blade-coated inside a nitrogen-filled glovebox. For the PM6 : DTY6 and PTQ10 : DTY6 blends, the solutions were stirred overnight at 70 °C before deposition.

For the device-area scaling experiment, we fixed the processing parameters corresponding to the final step of the upscaling procedure: fabrication outside the glovebox, a D/A ratio of 0.6 : 1.5, and *o*-xylene as the solvent. In this case, no gradient thicknesses were applied; instead, we prepared multiple samples with uniform active-layer thicknesses by varying the blade coating speed. For opaque devices, the coating speed ranged from 70 mm s^−1^ to 10 mm s^−1^, while for semitransparent devices, it ranged from 80 mm s^−1^ to 20 mm s^−1^. The electrodes were thermally evaporated following the same procedure previously described for both opaque and semitransparent configurations.

### 4.3 Module fabrication

Three types of modules were fabricated to investigate scalability and performance under varying conditions. The modules were based on the optimized active-layer (AL) blend of PTB7-Th : IEICO-4F with a 0.6 : 1.5 donor/acceptor (D/A) ratio and high-molecular-weight (125 kDa) PTB7-Th. The fabrication conditions and configurations for each module are detailed below:

#### 4.3.1 Opaque

The configuration consisted of glass/ITO/ZnO/AL/MoO_*x*_/Ag with 10 nm MoO_*x*_ and 100 nm Ag top electrodes. This module is composed of 4 subcells in series, with a total area of 2 cm^2^.

#### 4.3.2 ST (evaporated)

The configuration consisted of glass/ITO/ZnO/AL/MoO_*x*_/Ag with 10 nm MoO_*x*_ and 20 nm Ag top electrodes. This module is composed of 4 subcells in series, with a total area of 2 cm^2^.

The active layer was deposited using the same conditions optimized in the cell study. Laser patterning (P1, P2, and P3) was employed to create interconnections between subcells, enabling effective charge collection and module functionality. A schematic representation of the module interconnections is provided in Fig. S37. We used a Coherent EasyMark XL laser system operating at a wavelength of 1064 nm. For the P1 patterning step, we used a power of 13.2 W, a frequency of 80 kHz, a speed of 1000 mm s^−1^, and a pulse width of 8 ns. In the P2 step, the parameters varied based on the module type: for opaque modules, we used 9.2 W, 80 kHz, 4500 mm s^−1^, and a pulse width of 16 ns, while for ST modules, the parameters were 10 W, 80 kHz, 7000 mm s^−1^, and 16 ns. Finally, the P3 patterning step was performed with 11 W, 4000 kHz, 4000 mm s^−1^, and a pulse width of 2.5 ns.

For the opaque and evaporated semitransparent modules, vacuum evaporation was used for the MoO_*x*_ and Ag layers.

### 4.4 Solar-cell characterization

The *J*–*V* characteristics were measured in air using a Keithley 2400 source meter connected to an Arduino-based multiplexer/switcher, allowing the sequential measurement of up to 24 devices in less than 6 minutes. A SAN-EI Electric XES-AAA solar simulator supplied illumination and simulates AM1.5G conditions with homogeneous illumination across a 10 cm × 10 cm area. The intensity was calibrated at 100 mW cm^−2^ with a certified silicon solar-cell provided by Oriel.

### 4.5 Thin-film characterization: UV-vis spectroscopy

The optical properties were characterized using a UV-Vis/NIR spectrophotometer (V770, Jasco Inc.) for obtaining absorption curves, with measurements ranging from 300 to 1200 nm. The transmittance spectra for AVT determination were measured using a spectrometer (FLAME-S-VIS-NIR-ES, Ocean Optics) in combination with an integrating sphere.

### 4.6 Thin-film characterization: thickness

The active-layer thickness was measured using a mechanical profilometer (Dektak 150, Bruker). Thickness measurements were performed at three different points for each sample, and the average value calculated.

### 4.7 Thin-film characterization: GIWAXS

Grazing incidence wide-angle X-ray scattering (GIWAXS) experiments were performed at the BL11 NCD-SWEET beamline at the ALBA Synchrotron Radiation Facility, Spain (experiment ID AV-2024098865). The incident X-ray beam energy was set to 12.4 keV using a channel cut-Si (1 1 1) monochromator. The scattering patterns were recorded using a Rayonix® LX255-HS area detector, which consists of a pixel array of 1920 × 5760 pixels (*H* × *V*) with a pixel size of 44 × 44 µm^2^. Data are expressed as a function of the scattering vector (*q*), which was calibrated using Cr_2_O_3_ as standard sample to obtain a sample-to-detector distance of 204.84 mm. All images and linecuts were acquired at a pitch angle of 0.12°, corrected for the real-time synchrotron beam flux, the integration time (1 second), the silicon background (20% subtraction) and the film thickness. 2D GIWAXS patterns were corrected in pyFAI (Ewald's sphere correction) and plotted in the reciprocal space as a function of the radial (*q*_r_) and out-of-plane (*q*_z_) components of *q*. The in-plane (ip) and out-of-plane (oop) integrated linecuts of the 2D patterns were obtained after performing an azimuthal integration between 90° > *χ* > 45° and 45° > *χ* > 0°, respectively, *χ* being the azimuthal angle. The azimuthal quantitative analysis of the scattered intensity was performed by extracting and fitting the corresponding linecuts every 2° from *χ* = 1° to *χ* = 87°. However, due to the missing wedge derived from the Ewald's sphere correction, the usable linecuts and fits of the (010) reflections start at *χ* = 9°. The azimuthal linecuts were accordingly fitted using pseudo-Voigt functions together with a constant and an exponential decay function as the background, and the peak areas plotted as a function of *χ*. These were then fitted to Gaussian functions including, if needed, a constant background (*i.e.*, an isotropic population of crystallites). The resulting model function was finally corrected using the Lorentz factor sin(*χ*), and the area under the curve computed to quantify the amount of diffracting material in each case.

### 4.8 Thin-film characterization: Raman, PL and LBIC

The Raman scattering spectra and PL measurements performed in functional devices were acquired using a WITec alpha300 RA + confocal Raman setup, coupled to an Olympus objective with 10× magnification (NA 0.25). Two lasers centered at 633 and 785 nm were employed. The light was focused through the thick (1.1 mm), ITO-covered glass substrates and the laser power was between 3–5 mW for Raman and around 100 µW for PL measurements. All raw data were analysed using the WITec Project FIVE piece of software.

### 4.9 Charge mobility: space-charge-limited current (SCLC) method

Two device structures were fabricated for charge mobility analysis: an electron-only device with the configuration ITO/ZnO/AL/Ag and a hole-only device with ITO/BM-HTL-1/AL/MoO_*x*_/Ag. The AL was deposited with a thickness gradient, enabling a comprehensive study of charge transport properties across varying thicknesses, as well as easily finding the thickness values for which the space-charge regime is found.

The mobility values were extracted from the *J*–*V* curves obtained in the dark using the SCLC method. A Python script was developed to automate the analysis.

## Conflicts of interest

There are no conflicts to declare.

## Supplementary Material

TA-014-D5TA07234D-s001

TA-014-D5TA07234D-s002

## Data Availability

The data is contained within the paper in the supplementary information (SI). Supplementary information: the actual data from the manuscript, as well as a document with additional data, namely, the full device parameters for different thicknesses, compositions, molecular weight blends, modules, different donor/aceptor combinations, shelf lifetime, as well as complementary GIWAXS, absorption, LBIC and Raman data and analysis. See DOI: https://doi.org/10.1039/d5ta07234d.
