# Supplementary material for: Increasing polymer molecular weight enables low-donor-content, efficient and scalable semi-transparent organic solar cells
Source: J Mater Chem A Mater. 2026 Jan 27;14(22):13688–706. doi: 10.1039/d5ta07234d (PMC12937936; doi:10.1039/d5ta07234d)
Supplement: TA-014-D5TA07234D-s002 [file TA-014-D5TA07234D-s002.pdf]

Supplementary Information (SI) for Journal of Materials Chemistry A.
This journal is © The Royal Society of Chemistry 2026

Supporting Information

Increasing Polymer Molecular Weight Enables Low Donor Content, Efficient and Scalable Semi-Transparent Organic Solar Cells

Martín Martín-Ruiz[a], Paula Pinyol-Castillo[a,b,c], Xabier Rodríguez-Martínez[d], Jaime Martín[d] Ignasi Burgués-Ceballos[c], Mariano Campoy-Quiles[a,*].

[a]Institute of Materials Science of Barcelona (ICMAB-CSIC), Campus Universitat Autònoma de Barcelona, Bellaterra 08193, Spain.
[b]EURECAT Technology Centre of Catalonia.
[c]Institute of Energy Technologies, Department of Chemical Engineering, and Barcelona Research Center in Multiscale Science and Engineering, Universitat Politècnica de Catalunya.
[d]Centro de Investigación en Tecnologías Navales e Industriales (CITENI).

*Corresponding author.
E-mail: `mcampoy@icmab.es` (Mariano Campoy-Quiles)

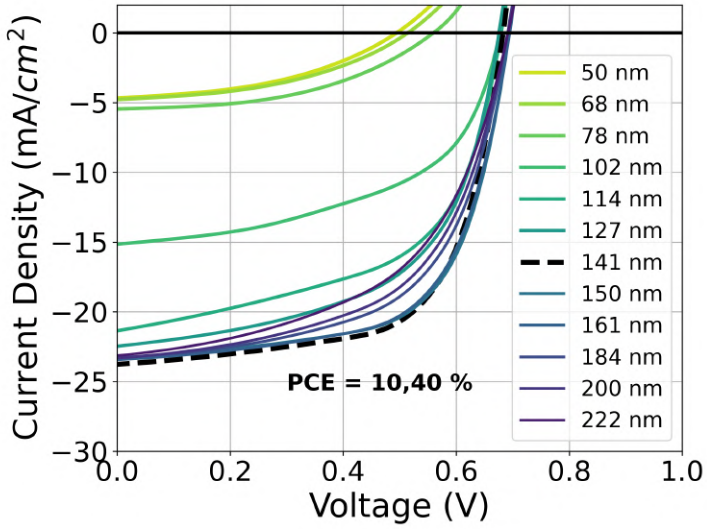

Figure S1. J-V characteristics of devices with 1.2:1.5 D/A ratio, corresponding to the points in Figure 1 d).

Absorption spectra

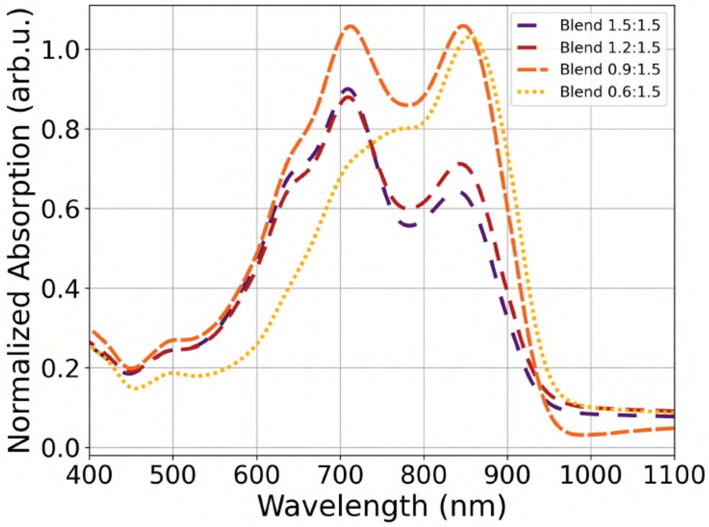

Figure S2. Absorption spectra of PTB7-TH and IEICO-4 blends with different D/A ratios.

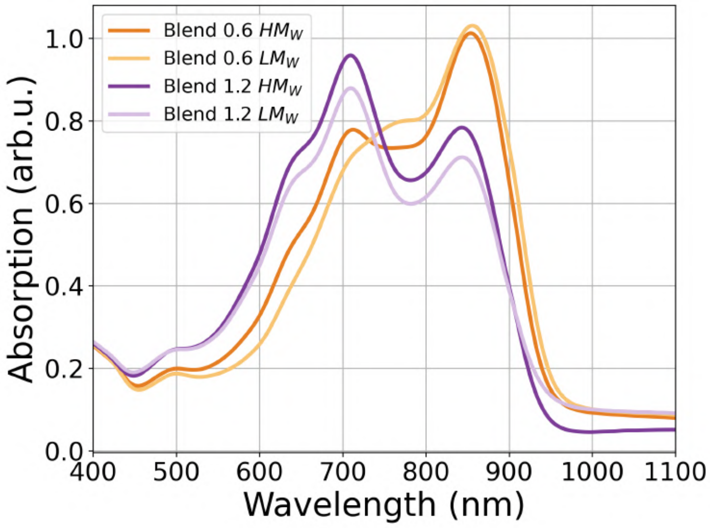

Figure S3. Absorption spectra of PTB7-TH and IEICO-4 blends with 0.6:1.5 and 1.2:1.5 D/A ratios with 125 kDa and 57 kDa molecular weight PTB7-TH.

D/A Ratio

Table S1. Fabrication yield for devices with High and Low Molecular Weight PTB7-Th at various donor ratios: The table shows the percentage of successfully fabricated devices for each combination of D/A ratio and molecular weight (M_w). Only devices fabricated using the same method and conditions are included, excluding samples from the broader upscaling study.

**D/A Ratio**	**0.4:1.5**	**0.4:1.5**	**0.6:1.5**	**0.6:1.5**	**1.0:1.5**	**1.0:1.5**	**1.2:1.5**	**1.2:1.5**	**1.5:1.5**	**1.5:1.5**
M_w **(kDa)**	High	Low	High	Low	High	Low	High	Low	High	Low
**Yield (%)**	63	21	83	71	80	80	73	67	91	25
**Total num. of devices**	24	24	48	48	24	24	72	72	24	24

Molecular Weight

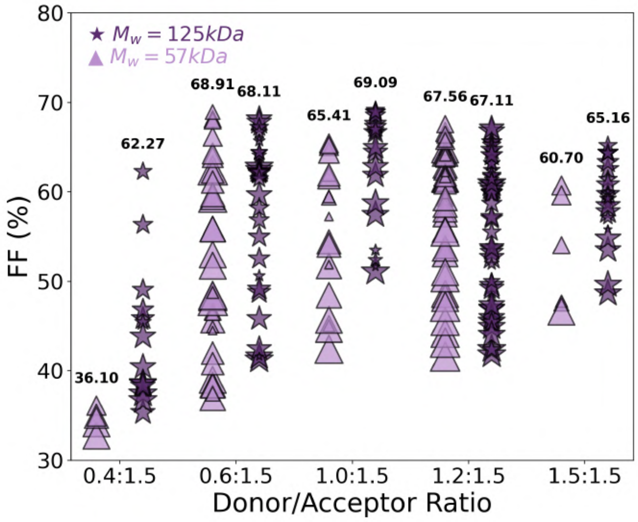

Figure S4. FF (%) and AL thickness (dif. size symbols) of organic solar cells with 57 kDa (triangles) and 125 kDa (stars) molecular weigth PTB7-TH at various D/A ratios in weight.

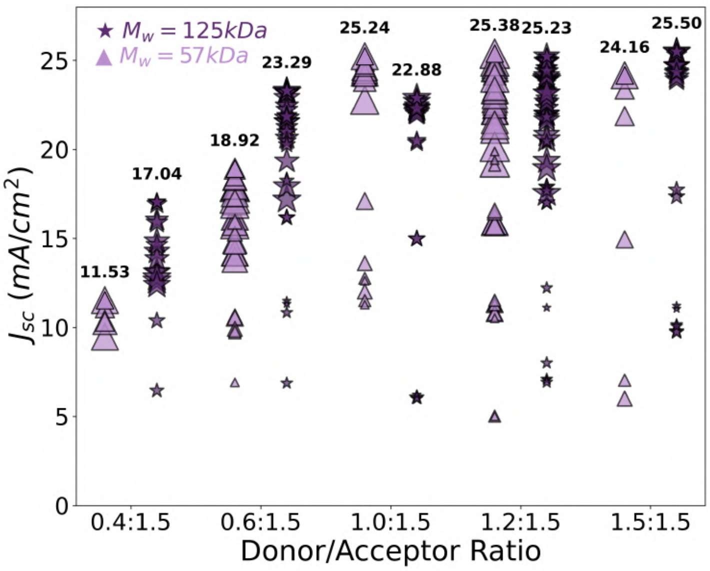

Figure S5. J_{sc} (mA/cm^2) and AL thickness (dif. size symbols) of organic solar cells with 57 kDa (triangles) and 125 kDa (stars) molecular weigth PTB7-TH at various D/A ratios in weight.

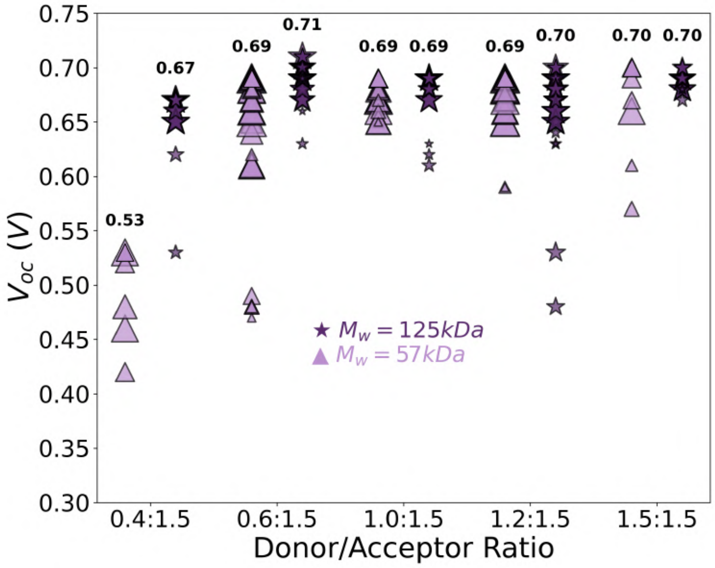

Figure S6. V_{oc} (V) and AL thickness (dif. size symbols) of organic solar cells with 57 kDa (triangles) and 125 kDa (stars) molecular weigth PTB7-TH at various D/A ratios in weight.

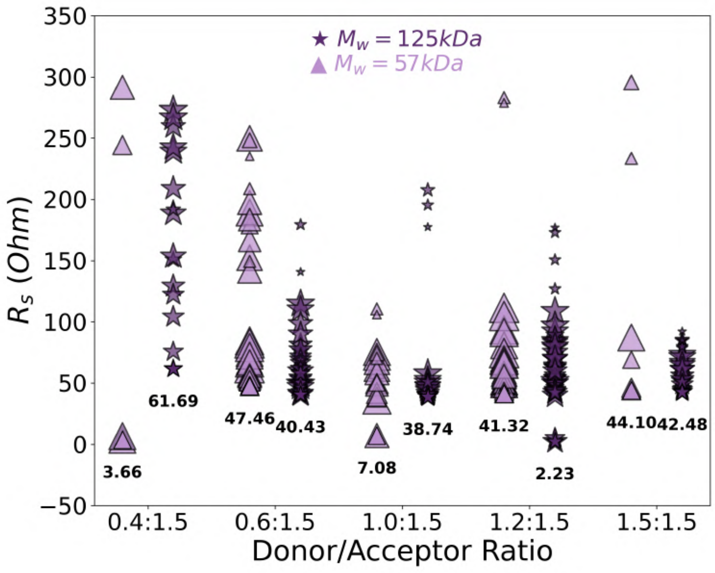

Figure S7. R_s (Ohm) and AL thickness (dif. size symbols) of organic solar cells with 57 kDa (triangles) and 125 kDa (stars) molecular weigth PTB7-TH at various D/A ratios in weight.

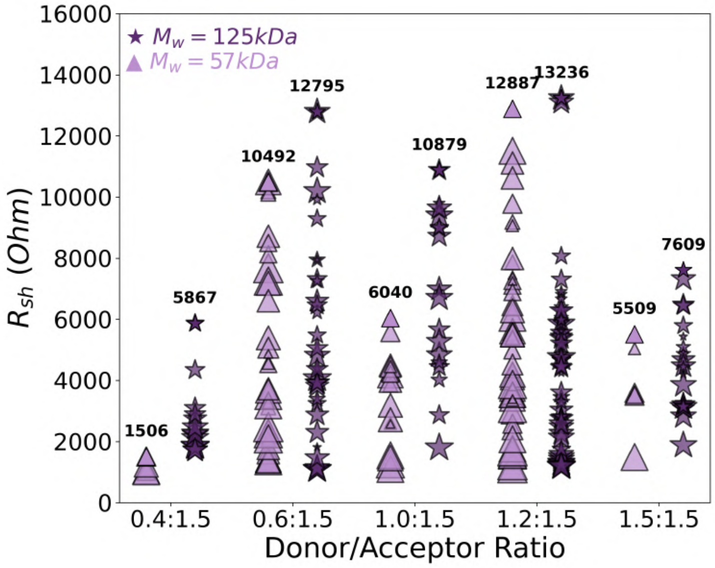

Figure S8. R_{sh} (Ohm) and AL thickness (dif. size symbols) of organic solar cells with 57 kDa (triangles) and 125 kDa (stars) molecular weigth PTB7-TH at various D/A ratios in weight.

Generality of Concepts

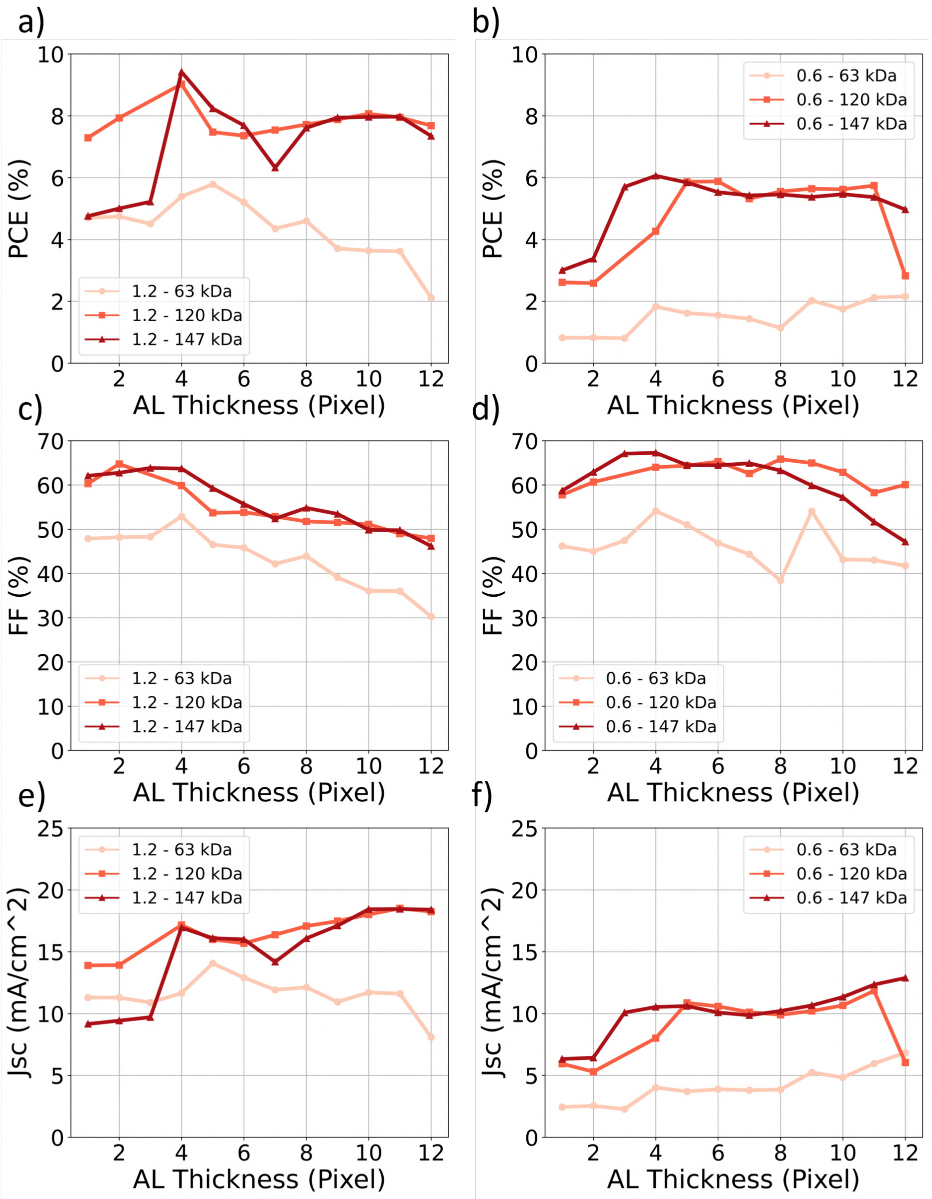

Figure S9. Photovoltaic parameters of PTQ10:DTY6 devices as a function of active layer thickness (pixel) for three donor molecular weights (63, 120 and 147 kDa) and two donor/acceptor ratios (1.2 and 0.6). Panels show the evolution of (a, b) PCE, (c, d) fill factor and (e, f) short-circuit current density for 1.2:1.5 D/A ratio (a, c, e) and 0.6:1.5 D/A ratio (b, d, f). Thickness increase with the number of pixels.

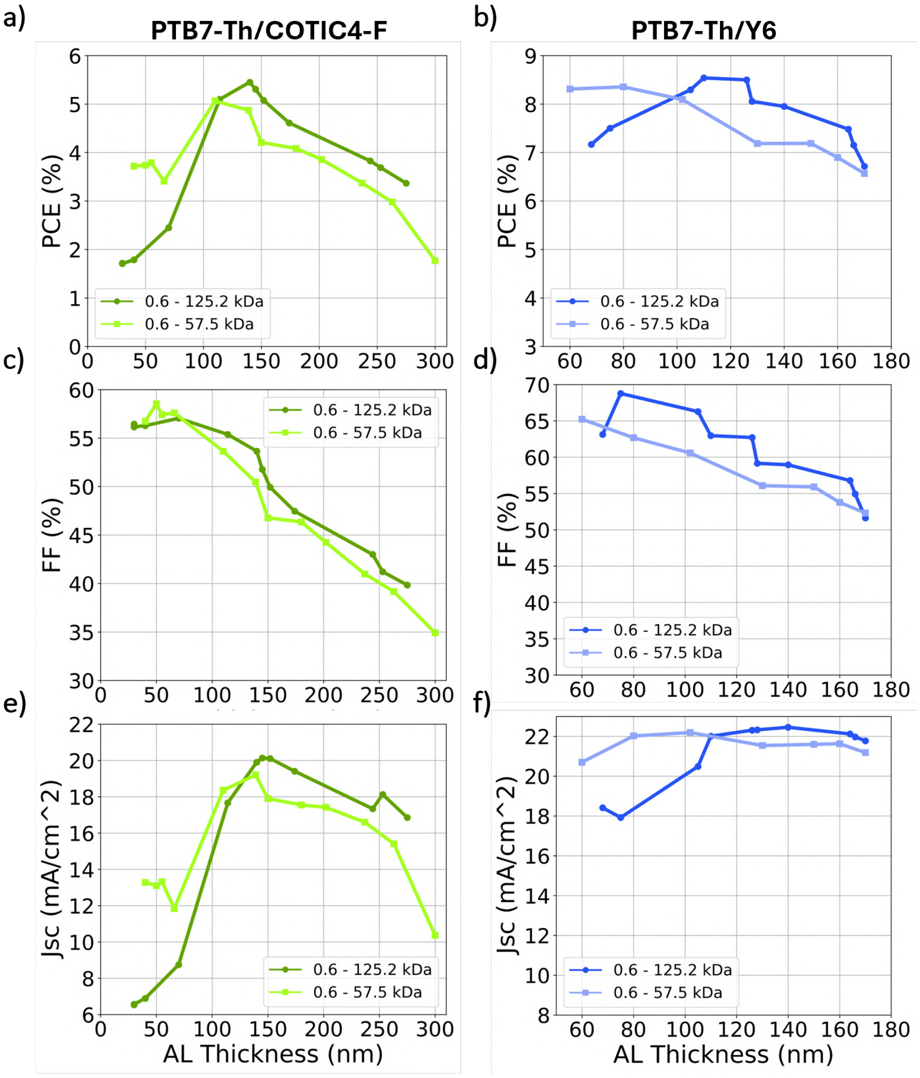

Figure S10. Photovoltaic parameters of PTB7-Th/COTIC-4F and PTB7-Th/Y6 devices as a function of active layer thickness (nm) for two donor molecular weights (57.5 and 125.2 kDa) and 0.6/1.5 donor/acceptor ratio. Panels show the evolution of (a, b) PCE, (c, d) fill factor and (e, f) short-circuit current density for COTIC-4F acceptor system (a, c, e) and Y6 acceptor system (b, d, f).

Shelf Lifetime Analysis

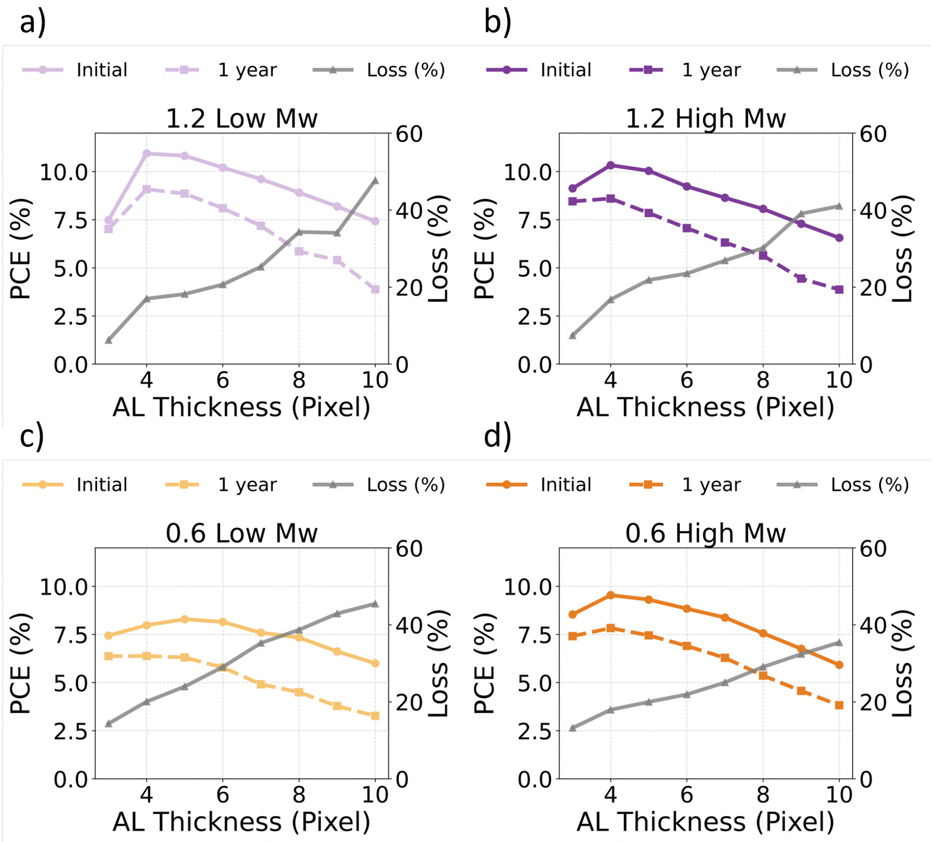

Figure S11. One-year ISOS-D-1 dark-storage stability of encapsulated PTB7-Th:IEICO-4F devices. Panels (a–d) show the evolution of PCE as a function of active layer (AL) thickness (Pixel) for the four representative cases: (a) 1.2 High Mw, (b) 0.6 High Mw, (c) 1.2 Low Mw and (d) 0.6 Low Mw. Solid lines correspond to the initial measurement and dashed lines to re-measure after 12 months of dark storage. Grey markers show the normalized stability loss (1-(PCE1y/PCE0)). Thickness increase with the number of pixels.

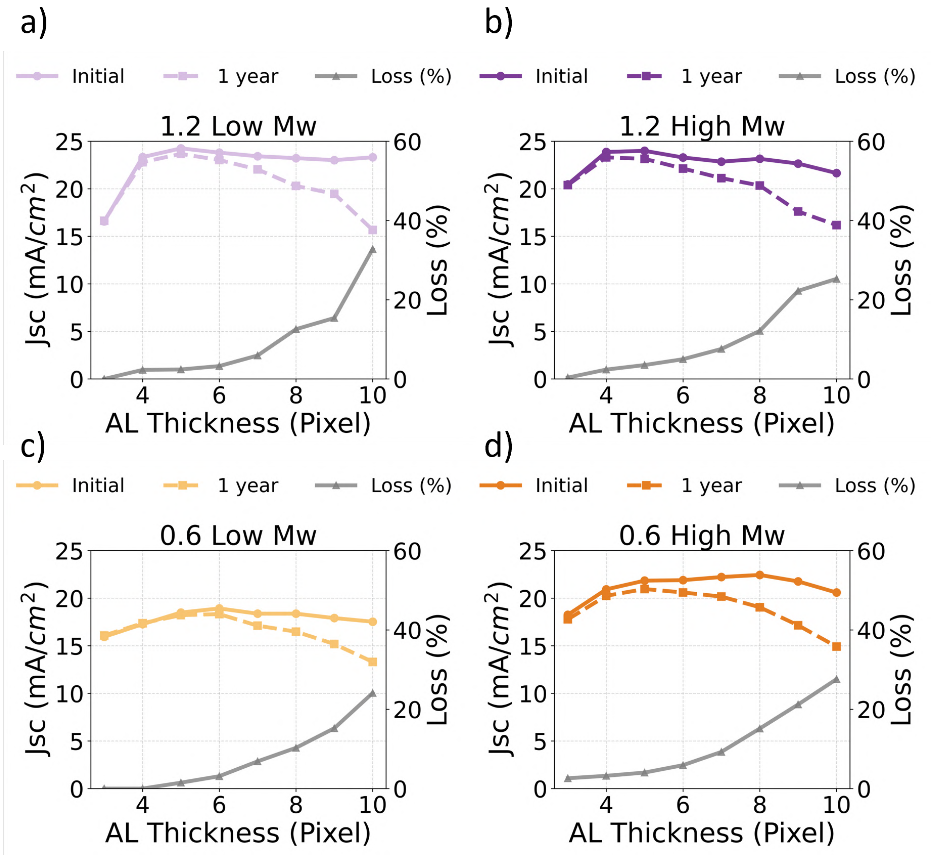

Figure S12. One-year ISOS-D-1 dark-storage stability of encapsulated PTB7-Th:IEICO-4F devices. Panels (a–d) show the evolution of J_{sc} as a function of active layer (AL) thickness (Pixel) for the four representative cases: (a) 1.2 High Mw, (b) 0.6 High Mw, (c) 1.2 Low Mw and (d) 0.6 Low Mw. Solid lines correspond to the initial measurement and dashed lines to re-measure after 12 months of dark storage. Grey markers show the normalized stability loss (1-(JSC1/Jsc0)). Thickness increase with the number of pixels.

Table S2. Electrical parameters (Voc, Jsc, FF and PCE) of the optimal pixel for each D/A ratio and molecular weight case before and after one year of ISOS-D-1 dark storage. The percentage loss relative to the fresh device is shown in parentheses next to each parameter. Losses are defined as (1-(Parameter1y / Parameter0)·100).

**Case**	**Donor Ratio**	**Mw (kDa)**	**Voc (V)**	**Jsc mA/cm2)**	**FF (%)**	**PCE (%)**
**Fresh**<br>**After 1y**	1.2	125	0.69<br>0.68 (1.0%)	23.5<br>23.31 (1.0%)	64.0<br>54.0 (15.6%)	10.3<br>8.6 (16.8%)
**Fresh**<br>**After 1y**	1.2	57	0.69<br>0.68 (0.8%)	23.3<br>22.8 (2.3%)	67.6<br>58.2 (13.9%)	10.9<br>9.0 (17%)
**Fresh**<br>**After 1y**	0.6	125	0.69<br>0.70 (-1.3%)	20.9<br>20.25 (3.2%)	65.7<br>55.3 (15.8%)	9.5<br>7.8 (17.9%)
**Fresh**<br>**After 1y**	0.6	57	0.69<br>0.66 (5.0%)	17.1<br>17.4 (-1.8%)	68.2<br>56.0 (17.7%)	8.0<br>6.4 (20.0%)

Grazing Incidence Wide-Angle X-ray Scattering (GIWAXS)

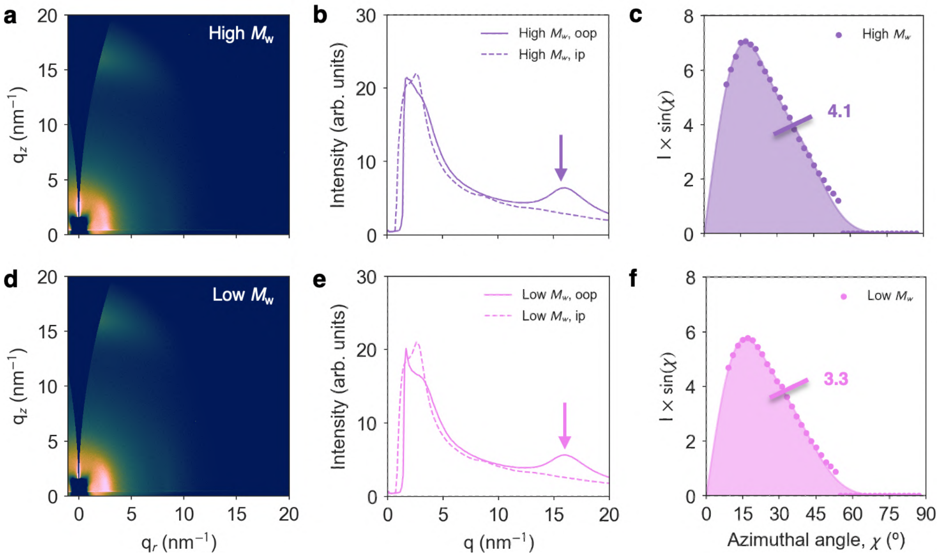

Figure S13. a),d) 2D GIWAXS diffractograms of neat PTB7-Th films of distinct molecular weight, as deposited on silicon. b),e) The corresponding out-of-plane and in-plane linecuts, with the arrows indicating the position of the (010) reflection. c), f) Azimuthal analysis of the scattering intensity of the (010) reflection, with the resulting area under the curve also indicated.

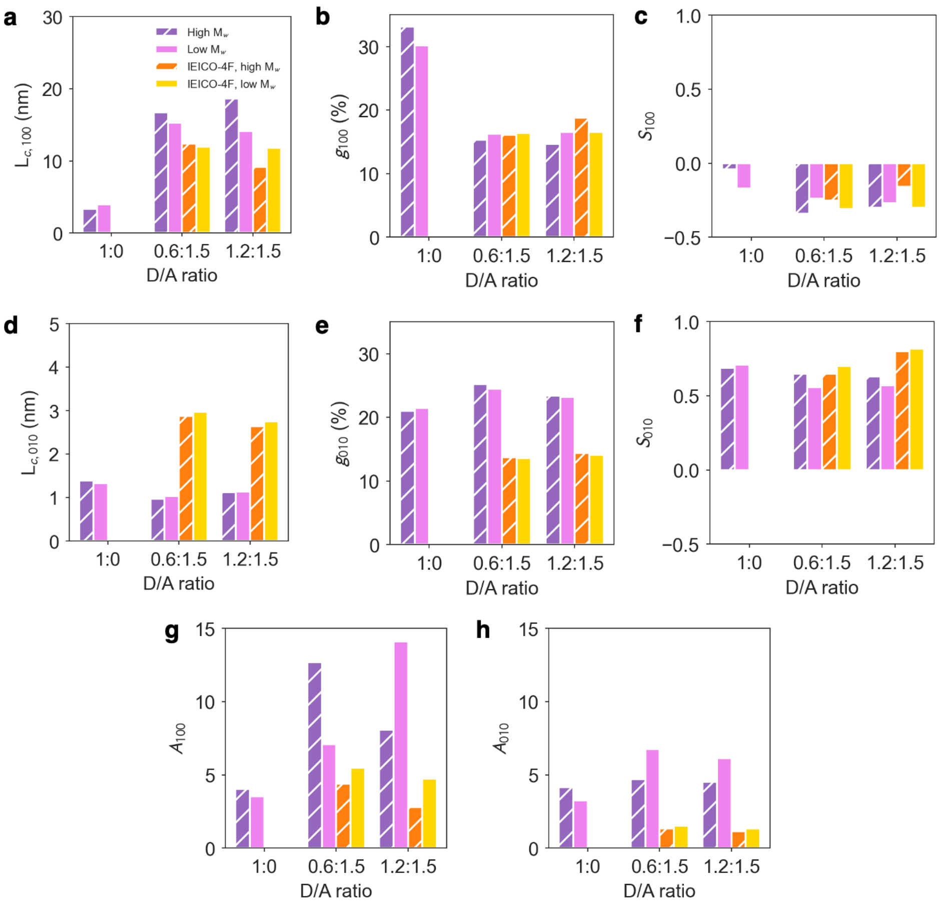

Figure S14. Graphical representation of the dataset included in Table R1, namely, Lc (a) and (d); g (b) and (e); S (c) and (f); and integrated (100) and (010) peak areas along the azimuthal angle (g) and (h).

Table S3. Collection of parameters derived from GIWAXS measurements describing the coherence length (L_c), the degree of paracrystallinity (g, %), the Hermans orientation parameter (S) and the area of the (010) and (100) peaks integrated azimuthally across the different D/A ratios and donor polymer molecular weights explored in this work. Note that L_c, g and S have been computed separately for D and A, and also for each characteristic peak (100) or (010).

**D/A ratio**	M_w **(kDa)**	**Material**	**Peak**	L_c **(nm)**	**g (%)**	**S**	A_{hkl}
**1:0**	57	PTB7-Th	(100)	4.0	30.2	-0.17	3.5
			(010)	1.3	21.5	0.71	3.3
	125	PTB7-Th	(100)	3.4	33.1	-0.04	4.0
			(010)	1.4	21.0	0.69	4.1
**0.6:1.5**	57	PTB7-Th	(010)	1.0	24.5	0.56	6.8
		IEICO-4F	(010)	3.0	13.6	0.7	1.5
		PTB7-Th	(100)	15.3	16.2	-0.24	7.1
		IEICO-4F	(100)	12.0	16.4	-0.31	5.5
	125	PTB7-Th	(010)	1.0	25.2	0.65	4.7
		IEICO-4F	(010)	2.9	13.8	0.65	1.3
		PTB7-Th	(100)	16.7	15.3	-0.34	12.7
		IEICO-4F	(100)	12.4	16.1	-0.25	4.4
**1.2:1.5**	57	PTB7-Th	(010)	1.1	23.2	0.57	6.1
		IEICO-4F	(010)	2.8	14.1	0.82	1.3
		PTB7-Th	(100)	14.1	16.5	-0.27	14.1
		IEICO-4F	(100)	11.8	16.5	-0.30	4.7
	125	PTB7-Th	(010)	1.1	23.4	0.63	4.5
		IEICO-4F	(010)	2.6	14.4	0.80	1.2
		PTB7-Th	(100)	18.6	14.7	-0.30	8.1
		IEICO-4F	(100)	9.2	18.8	-0.16	2.8

Transparency

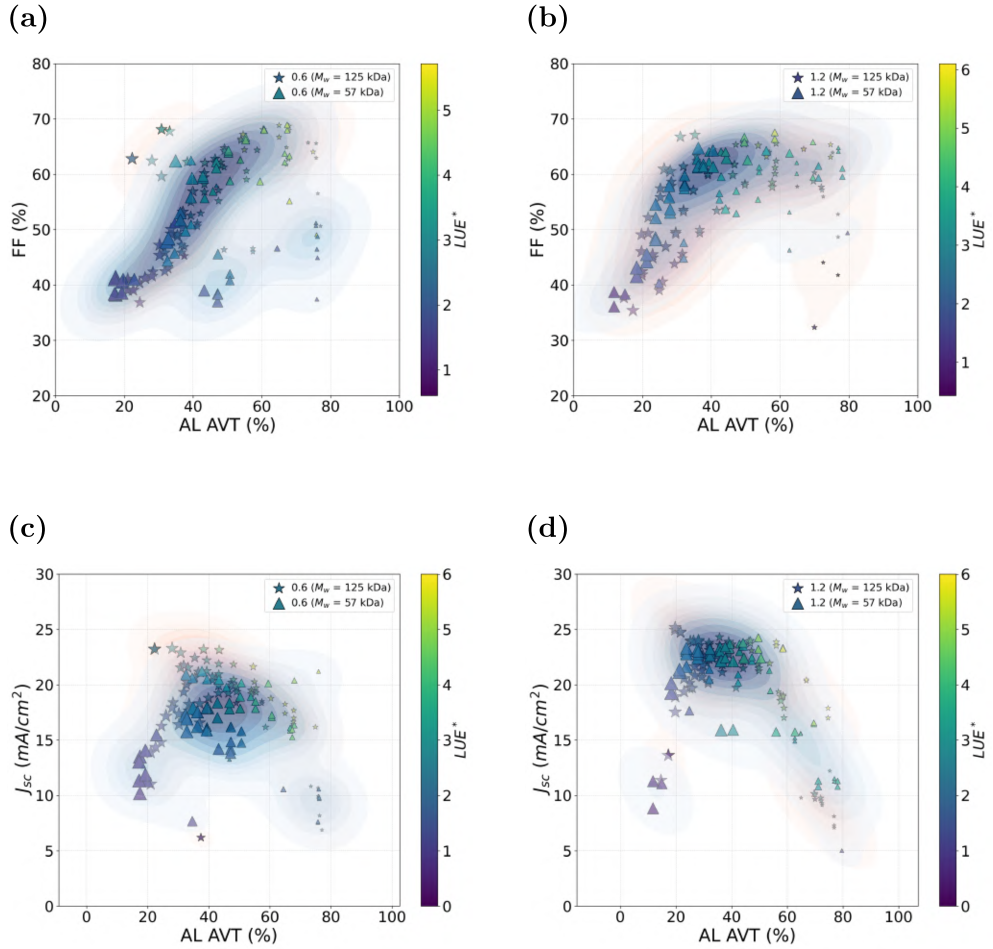

Figure S15. Fill factor (FF) and Light Utilization Efficiency (LUE*) as a function of active layer (AL) average visible transmittance (AVT) for devices with PTB7-Th/IEICO-4F at **a)** 0.6:1.6 and **b)** 1.2:1.6. J_{sc} and LUE* as a function of AL AVT for devices with PTB7-Th/IEICO-4F at **c)** 0.6:1.6 and **d)** 1.2:1.6 D/A ratio.

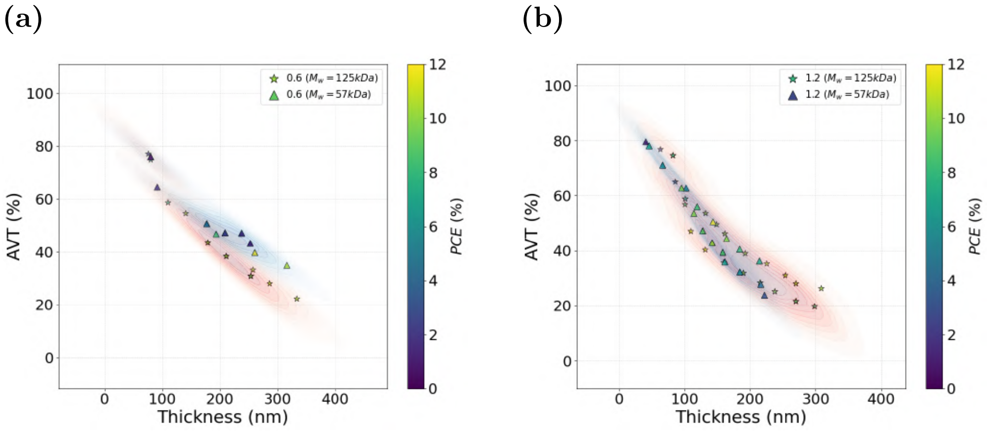

Figure S16. AVT and Power Conversion Efficiency (PCE) as a function of AL thickness for devices with PTB7-Th/IEICO-4F at **a)** 0.6:1.6 and **b)** 1.2:1.6 D/A ratio.

Intermediate Molecular Weight

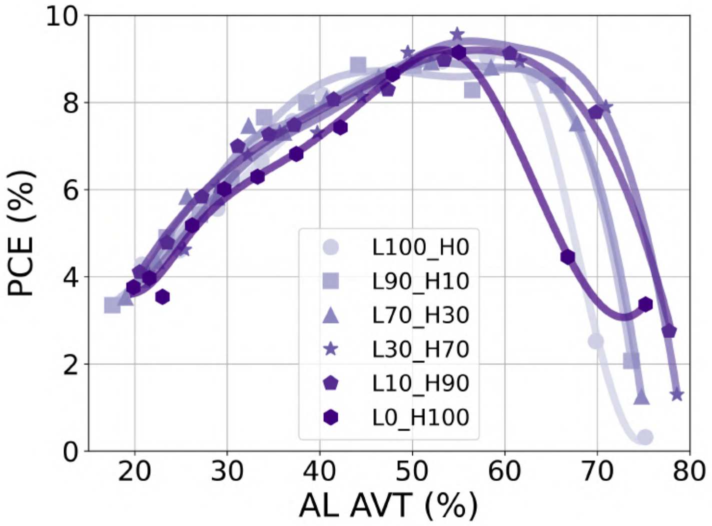

Figure S17. PCE (%) as a function of active layer average visual transmission for samples with different molecular weight donor composition.

Upscaling

Table S4. Fabrication yield for devices with High and Low Molecular Weight PTB7-Th at various donor ratios: The table shows the percentage of successfully fabricated devices for each combination of D/A ratio and molecular weight (M_w). All devices fabricated using different method and conditions from upscaling study are included

D_R	**0.6**	**0.6**	**1.2**	**1.2**
M_w **(kDa)**	High	Low	High	Low
**Yield (%)**	65	50	66	67
**Total num. of devices**	384	120	216	96

Additive content

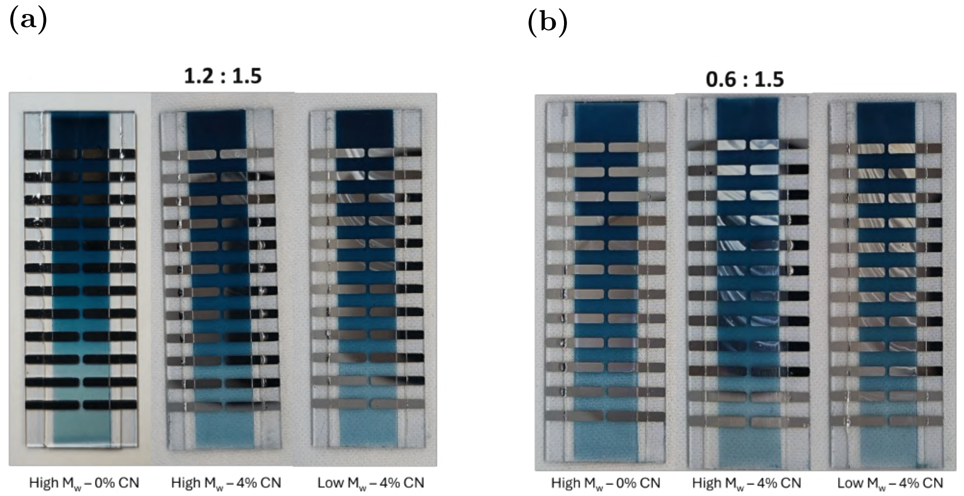

Figure S18. a) Diagram of the samples fabricated with different D/A ratios, M_w and additive content.

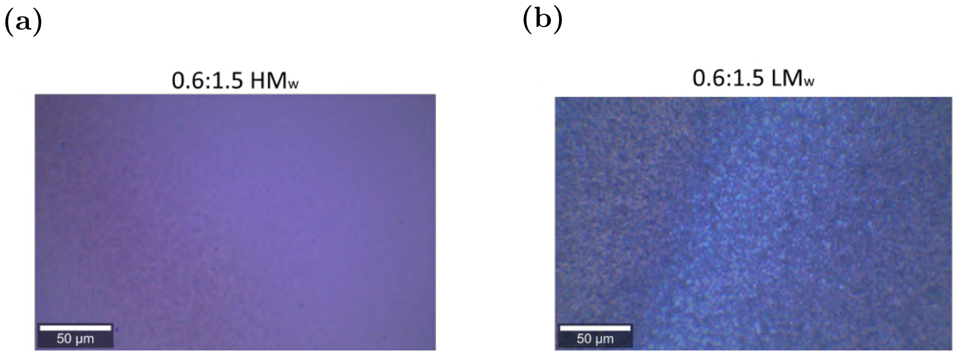

Figure S19. Microscopy image (40×) showing white stains in the active layer for a) 125 kDa and b) 57 kDa PTB7-TH M_w at 0.6:1.5 D/A ratio.

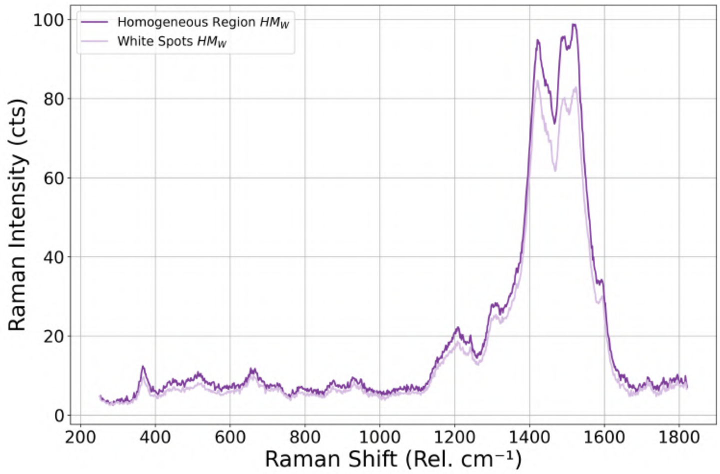

Figure S20. Raman spectra comparison between the white spots regions with the normal active layer for a 0.6:1.5 D/A ratio and the higher M_w PTB7-TH

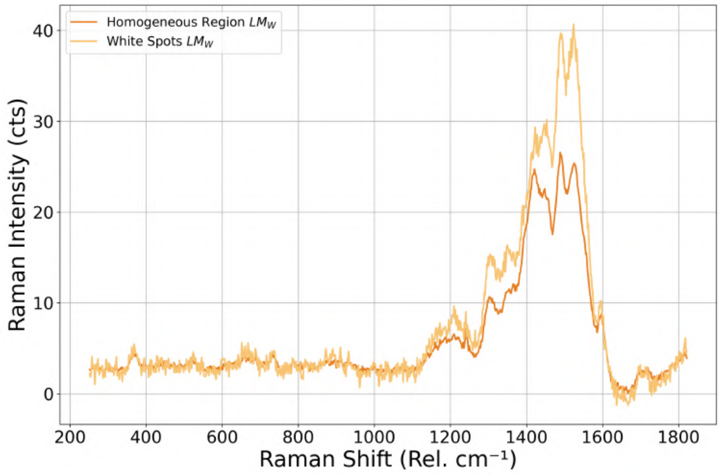

Figure S21. Raman spectra comparison between the white spots regions with the normal active layer for a 0.6:1.5 D/A ratio and the lower M_w PTB7-TH

Semitransparent Electrodes

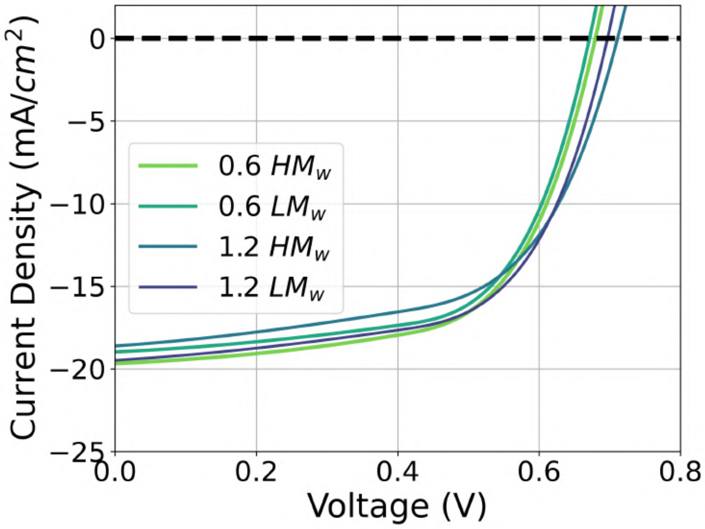

Figure S22. *J*-*V* characteristics of semitransparent devices in Table 4.

Table S5. Summary of the photovoltaic performance (PCE), average visible transmittance (AVT), and light utilization efficiency (LUE) of the best-performing semitransparent device from this study, compared with representative literature values for PTB7-Th:IEICO-4F based systems. Full-stack values are reported for consistency. Key fabrication parameters—such as active layer thickness, solvent, processing method, electron transport layer (ETL), hole transport layer (HTL), TE (top electrode) materials and device area are also included for reference. LBL (layer by layer deposition), APT (average plant transmission).

Active Layer Thickness (nm)	Solvent	Fabrication Method	Substrate	ETL	HTL	Top Electrode	Area (mm^2)	PCE (%)	AVT (%)	LUE (%)	Anti-reflective	Ref.
x	O-xylene	blade-coating	Glass	ZnO	MoOx (15nm)	Au (1 nm)<br>Ag (20 nm)	8.0	8.2	25.8	2.1	No	This work
x	CN/CB	spin-coating	Glass	ZnO (30nm)	MoOx (10nm)	Au (1 nm)<br>Ag (25 nm)	10.0	9.6	32.6	3.1	No	1
x	CB<br>1,8-diiodooctane	spin-coating	Glass	ZnO	MoOx (7nm)	Au (1 nm)<br>Ag (10 nm)	4.0	9.6	28.1	2.7	No	2
100	CB:1-CN	spin-coating	Glass	SnO3 (10nm)	MoOx (10nm)	Ag (15 nm)	4.0	8.7	25.1	2.2	No	3
x	C6H5Cl	spin-coating	Glass	PEDOT:PSS	PDIN	Au (1 nm)<br>Ag (10 nm)	3.8	9.4	24.6	2.3	No	4
60	CB	spin-coating	Glass	ZnO	WO3 (30nm)	Ag (15nm)	4.6	7.0	46.7 (APT)	3.3	Yes	5
110	CB:DIO	spin-coating	Glass	PEDOT:PSS	PFN-Br (sc)	Ag (20nm)	5.2	10.8	29.5	3.2	Yes	6
PTB7-Th(40nm)<br>IEICO-4F (55nm)<br>LBL	PTB7-Th<br>o-xylene<br>IEICO-4F<br>o-xylene<br>n-butanol<br>1-chloronaphthalene	spin-coating	Glass	ZnO	MoO3 (7nm)	Ag (12 nm)	4.4	8.4	41.6	3.5	Yes	7
PTB7-Th(60nm)<br>IEICO-4F (70nm)<br>LBL	PTB7-Th<br>o-xylene<br>IEICO-4F<br>o-xylene<br>n-butanol<br>1-chloronaphthalene	spin-coating	Glass	PEDOT:PSS (40nm)	PNDIT-F3N-Br (5nm)	Au (1 nm)<br>Ag (12 nm)	4.0	8.3	21.0	1.7	No	8
110	CB	spin-coating	Glass	ZnO	PEDOT:PSS (30nm)	AgNWs-BM	6.9	7.1	33.0	2.4	No	9
x	CB	spin-coating	PET	ZnO	MoOx (10nm)	Au (1 nm)<br>Ag (15 nm)	100.0	10.0	34.2	3.4	No	10

Area

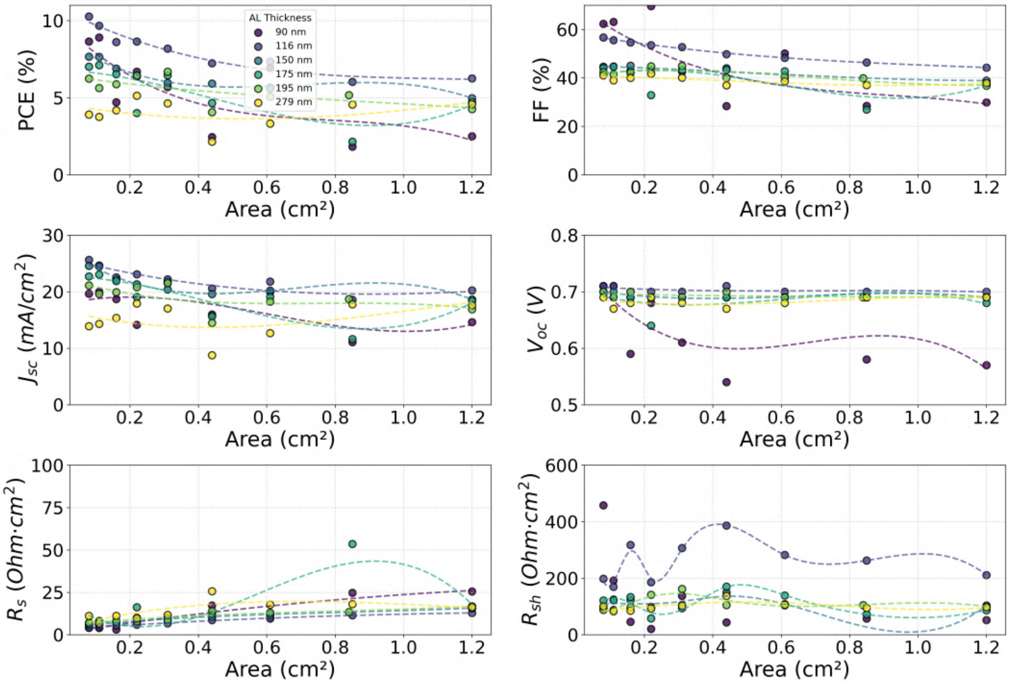

Figure S23. PCE (%), FF (%), V_{oc} (V), J_{sc} (mA/cm^2), R_s (Ohm·cm^2) and R_sh (Ohm·cm^2) of devices as a function of device area for opaque configurations with different AL thicknesses.

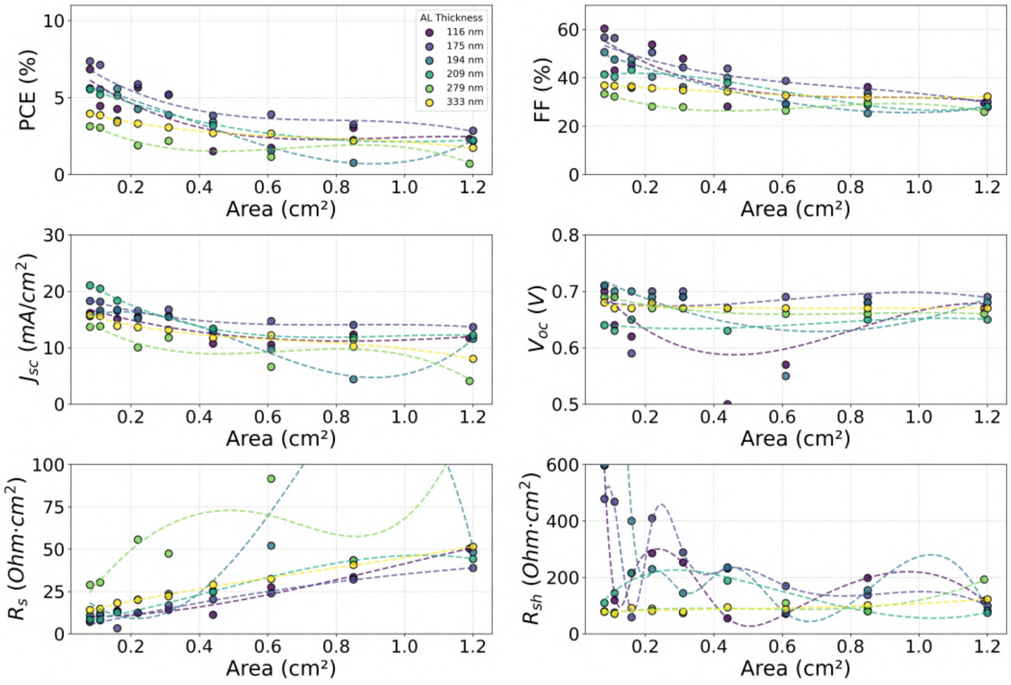

Figure S24. PCE (%), FF (%), V_{oc} (V), J_{sc} (mA/cm^2), R_s (Ohm·cm^2) and R_sh (Ohm·cm^2) of devices as a function of device area for semitransparent configurations with different AL thicknesses.

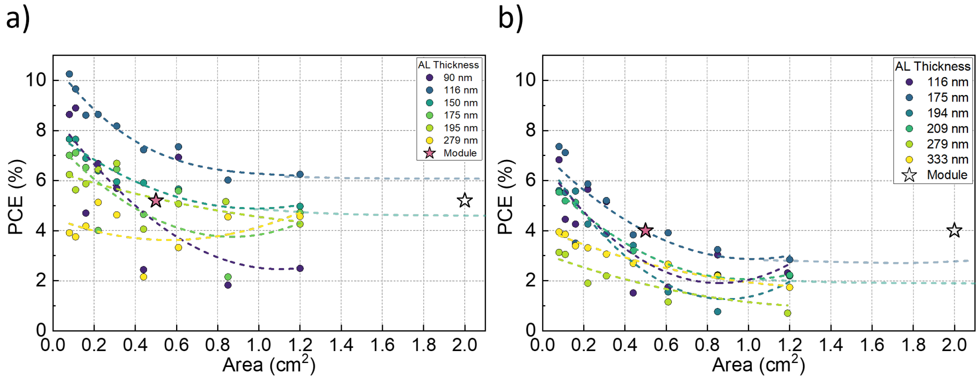

Figure S25. PCE (%) of devices as a function of device area for opaque (a) and semitransparent (b) configurations with different AL thicknesses. The filled star corresponds to the module PCE plotted at the area of a single module subcell (0.5 cm^2), while the open star represents the PCE of the full module (2 cm^2).

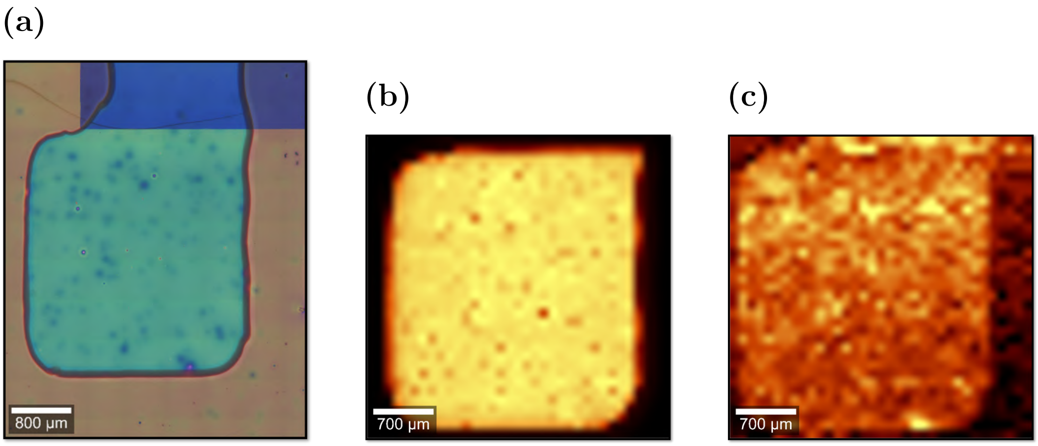

Figure S26. a)Microscopy 10x, b) light beam induced current (LBIC) and c) Photoluminescence (PL) images of an 8 mm^2 pixel.

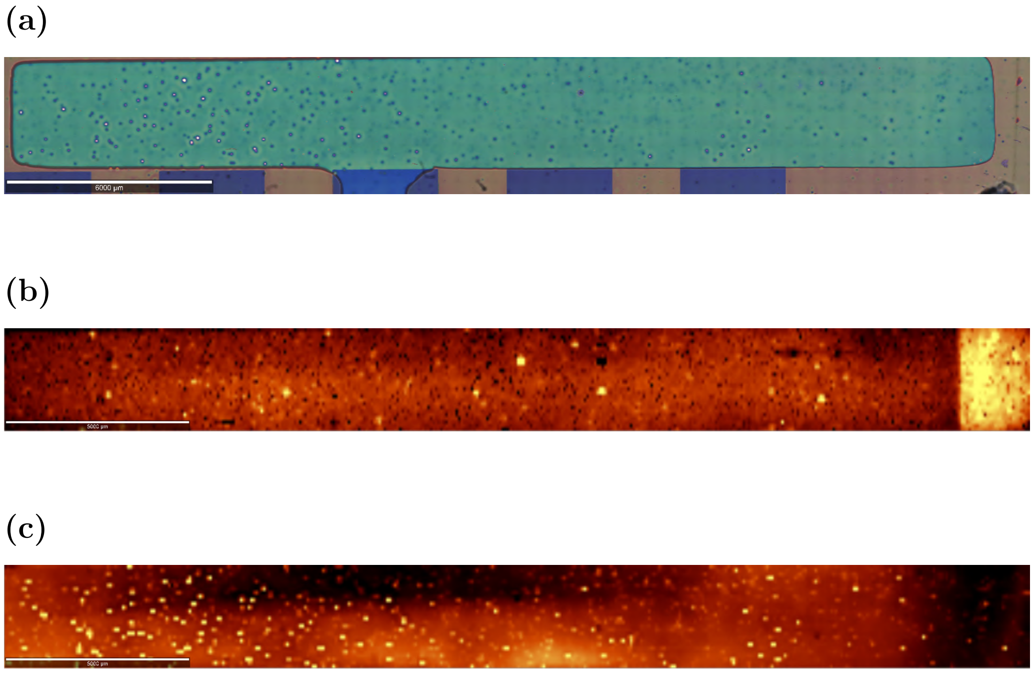

Figure S27. a)Microscopy 10x, b) LBIC and c) PL images of an 60 mm^2 pixel.

Raman

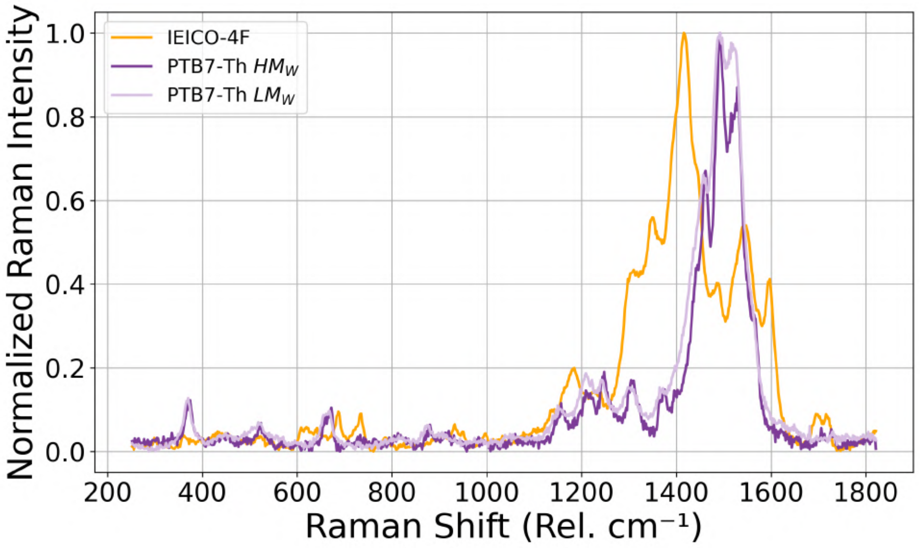

Figure S28. Raman spectra of the reference materials: PTB7-TH (125 kDa), PTB7-TH (57 kDa) and IEICO-4F

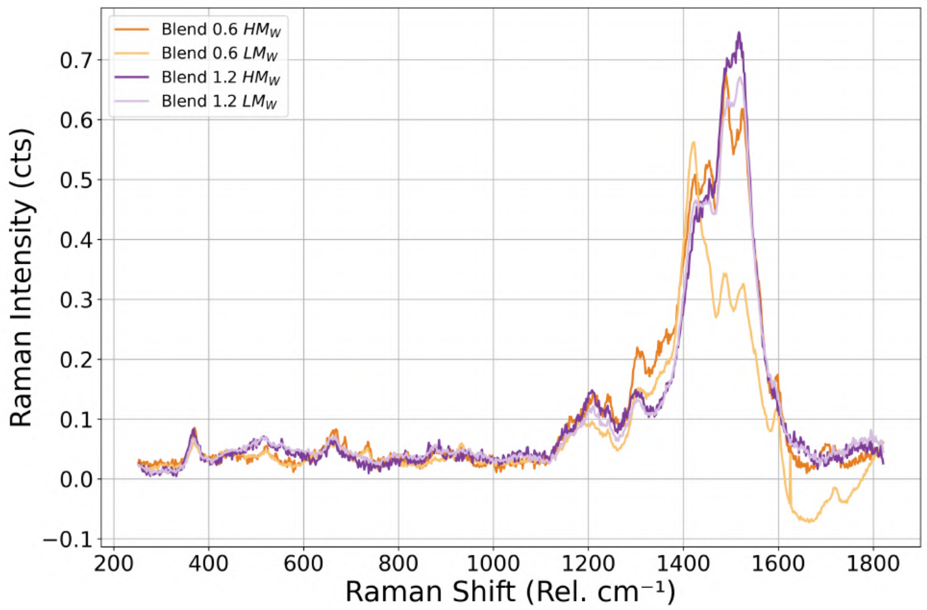

Figure S29. Raman spectra comparison between active layer blends with 125 kDa and 57 kDa M_w PTB7-TH for a 0.6:1.5 and 1.2:1.5 D/A ratio

PL

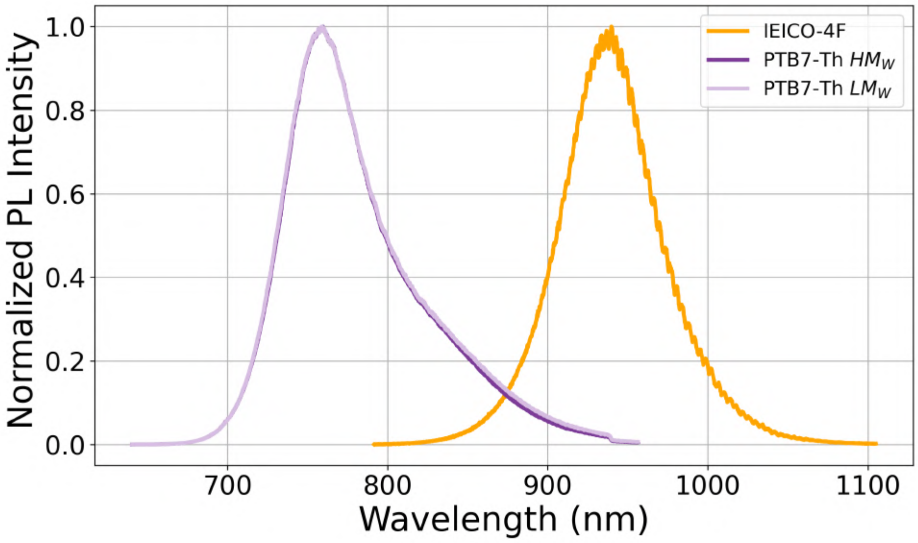

Figure S30. PL spectra of the reference materials: PTB7-TH (125 kDa), PTB7-TH (57 kDa) and IEICO-4F

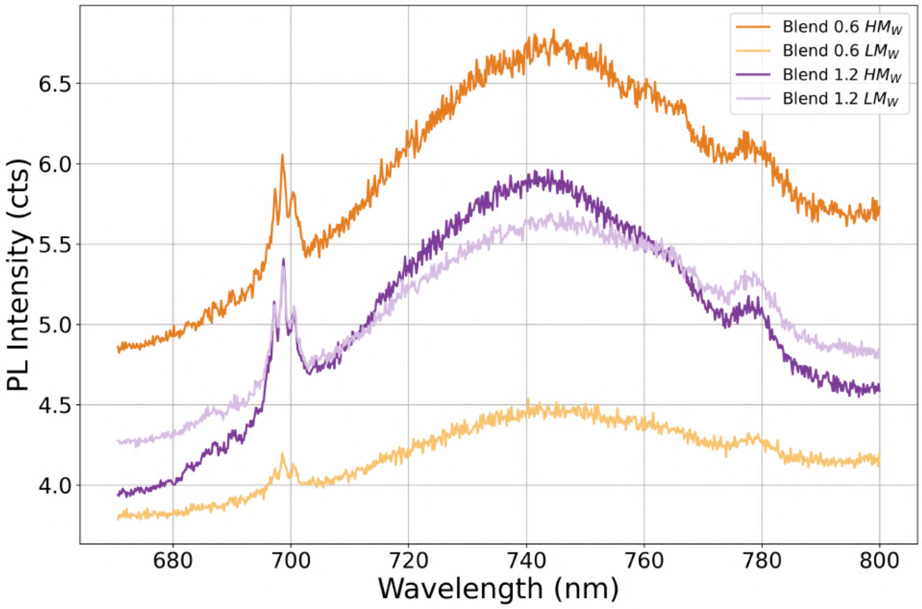

Figure S31. PL spectra comparison between active layer blends with 125 kDa and 57 kDa M_w PTB7-TH for a 0.6:1.5 and 1.2:1.5 D/A ratio. Excitation was performed using a 633 nm laser.

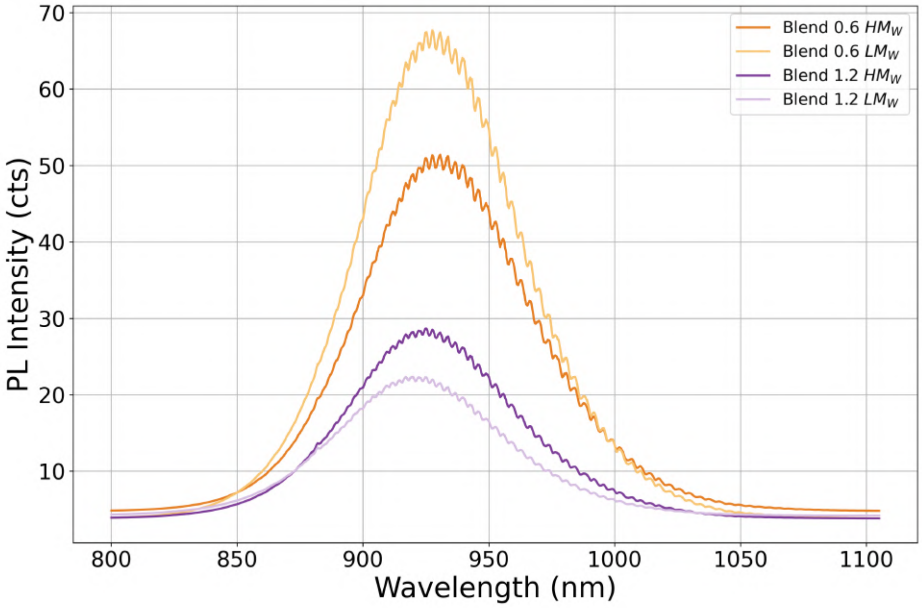

Figure S32. PL spectra comparison between active layer blends with 125 kDa and 57 kDa M_w PTB7-TH for a 0.6:1.5 and 1.2:1.5 D/A ratio. Excitation was performed using a 785 nm laser.

Defects

Table S6. Defects density for different D/A ratios, molecular weights, and CN additive contents. The values were obtained by analyzing microscopy images (10×) of four pixels (two thick and two thin) per sample.

**D/A Ratio**	**Add (%)**	M_w	**Thickness (nm)**	**Number of Defects**	**Defects Density** (def/mm^2)	**Defects Density** $\cdot 10^4$ (def/mm^3)
0.6:1.5	4	High	250	175	22	9
0.6:1.5	4	High	140	128	16	12
0.6:1.5	4	Low	240	108	14	6
0.6:1.5	4	Low	140	122	16	11
0.6:1.5	0	High	250	106	14	5
0.6:1.5	0	High	140	96	12	9
1.2:1.5	4	High	225	503	64	29
1.2:1.5	4	High	130	74	9	7
1.2:1.5	4	Low	184	696	89	48
1.2:1.5	4	Low	100	135	17	17
1.2:1.5	0	High	225	885	113	50
1.2:1.5	0	High	130	362	46	36

(a) (b) (c)

(d) (e) (f)

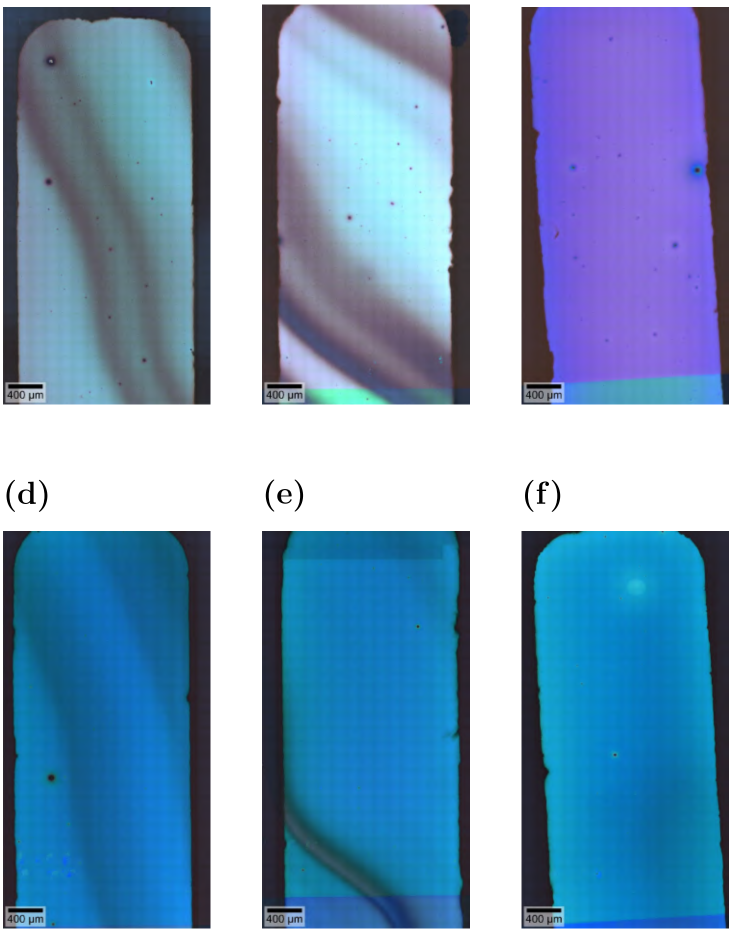

Figure S33. Microscopy images (10×) of six representative pixels for different processing conditions and 0.6:1.5 D/A ratio: (a) High M_w, 4% additive, Thick AL. (b) Low M_w, 4% additive, Thick AL. (c) High M_w, 0% additive, Thick AL. (d) High M_w, 4% additive, Thin AL. (e) Low M_w, 4% additive, Thin AL. (f) High M_w, 0% additive, Thin AL.

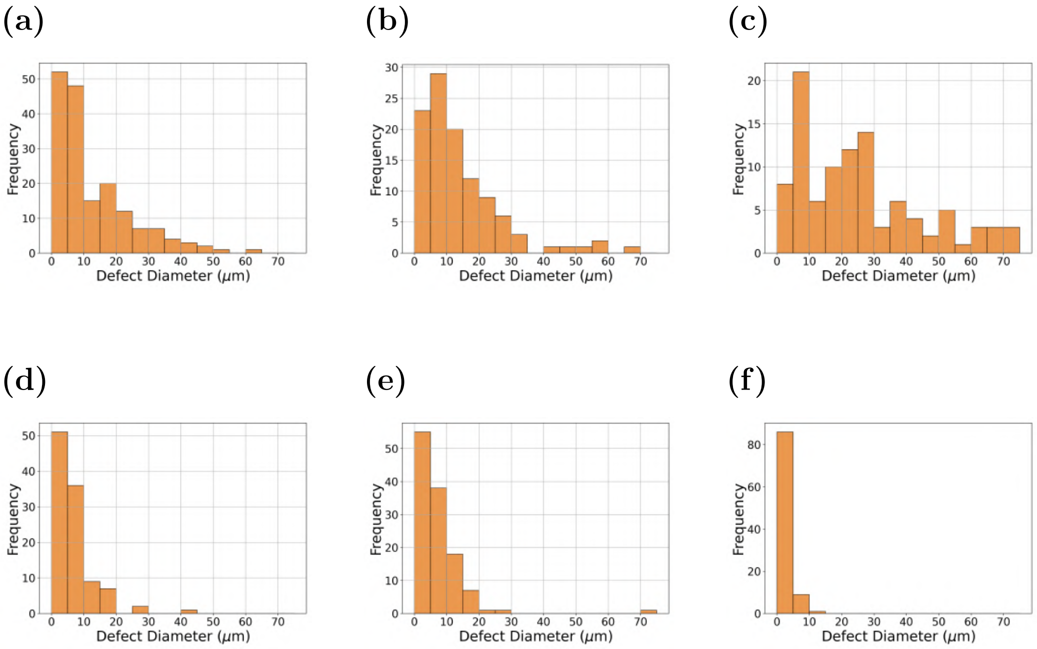

Figure S34. Defect size distribution of the pixels for different processing conditions and 0.6:1.5 D/A ratio: (a) High M_w, 4% additive, Thick AL. (b) Low M_w, 4% additive, Thick AL. (c) High M_w, 0% additive, Thick AL. (d) High M_w, 4% additive, Thin AL. (e) Low M_w, 4% additive, Thin AL. (f) High M_w, 0% additive, Thin AL.

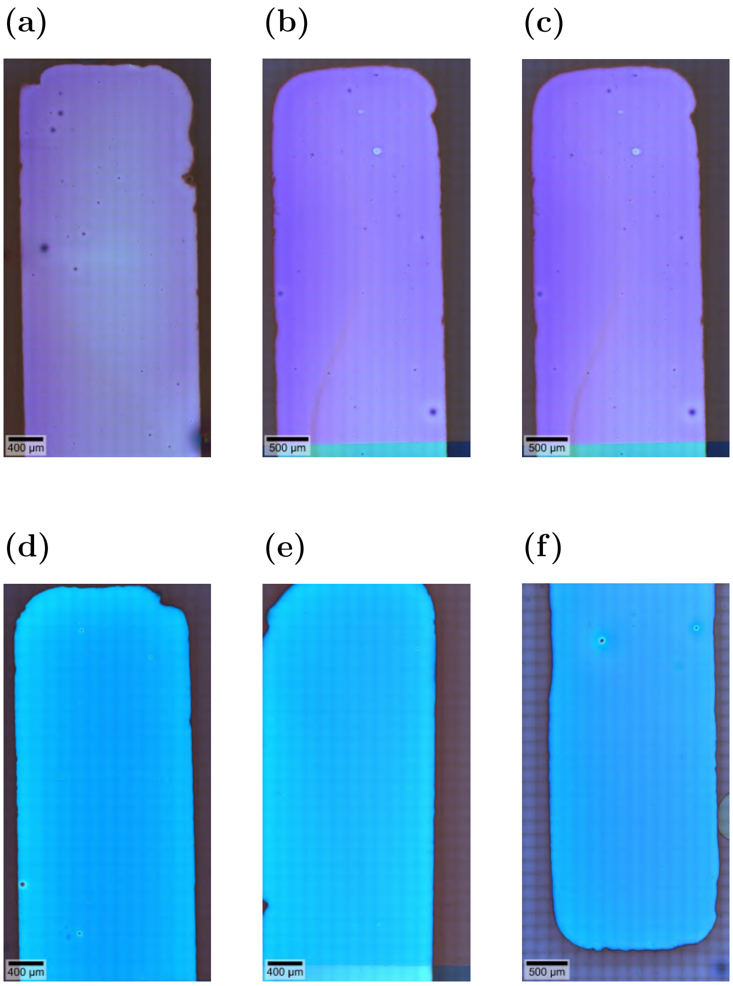

Figure S35. Microscopy images (10×) of six representative pixels for different processing conditions and 1.2:1.5 D/A ratio: (a) High M_w, 4% additive, Thick AL. (b) Low M_w, 4% additive, Thick AL. (c) High M_w, 0% additive, Thick AL. (d) High M_w, 4% additive, Thin AL. (e) Low M_w, 4% additive, Thin AL. (f) High M_w, 0% additive, Thin AL.

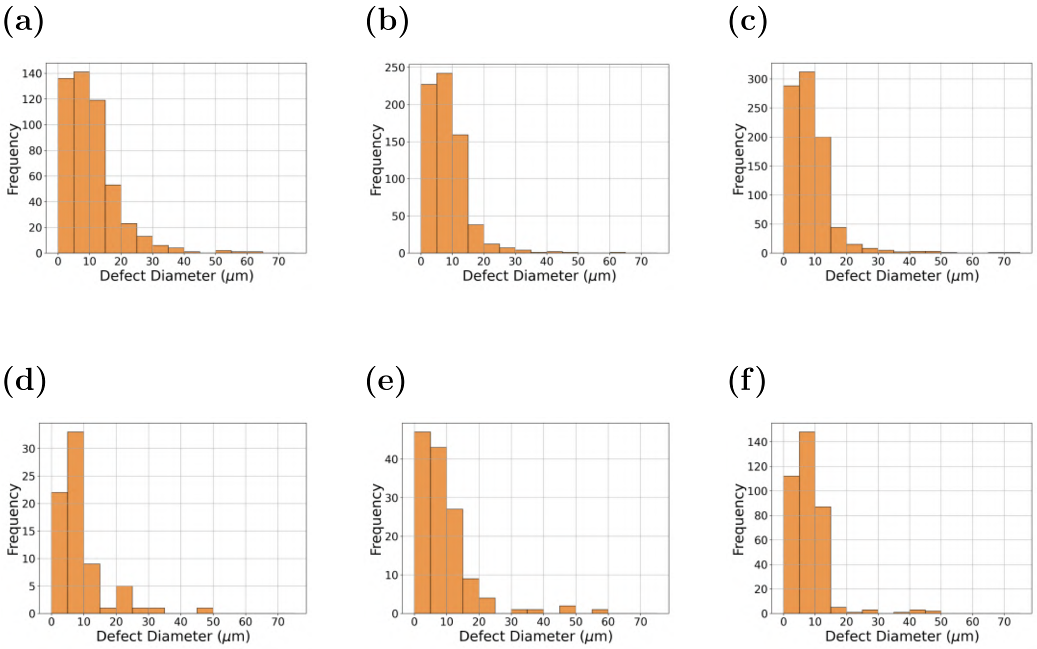

Figure S36. Defect size distribution of the pixels for different processing conditions and 1.2:1.5 D/A ratio: (a) High M_w, 4% additive, Thick AL. (b) Low M_w, 4% additive, Thick AL. (c) High M_w, 0% additive, Thick AL. (d) High M_w, 4% additive, Thin AL. (e) Low M_w, 4% additive, Thin AL. (f) High M_w, 0% additive, Thin AL.

Modules

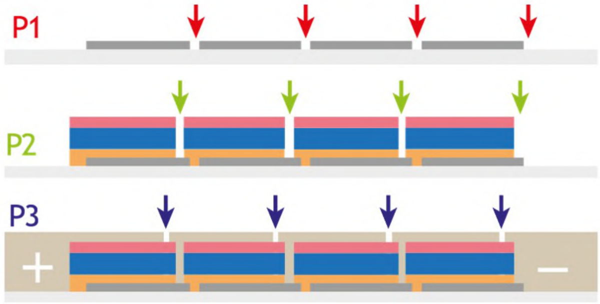

Figure S37. Schematic of module interconnections illustrating the laser-patterning process. Materials are color-coded as follows: Light grey-Glass substrate; Dark grey-Bottom electrode (ITO); Yellow-Electron Transport Layer (ETL); Blue-Active Layer; Pink-Hole Transport Layer (HTL); Brown-Top electrode (Ag).

References

1. Cheng, Pei, et al. "Transparent hole-transporting frameworks: a unique strategy to design high-performance semitransparent organic photovoltaics." Advanced Materials 32.39 (2020): 2003891.

2. Li, Yuanhao, et al. "Optical and electrical losses in semitransparent organic photovoltaics." Joule 8.2 (2024): 527-541.

3. Bai, Yiming, et al. "Interfacial engineering and optical coupling for multicolored semitransparent inverted organic photovoltaics with a record efficiency of over 12%." Journal of Materials Chemistry A 7.26 (2019): 15887-15894.

4. Hu, Zhenghao, et al. "Semitransparent ternary nonfullerene polymer solar cells exhibiting 9.40% efficiency and 24.6% average visible transmittance." Nano Energy 55 (2019): 424-432.

5. Oh, Juhui, et al. "Photopically transparent organic solar cells with tungsten oxide-based multilayer electrodes." ACS Applied Materials & Interfaces 15.36 (2023): 42802-42810.

6. Xia, Ruoxi, et al. "High-throughput optical screening for efficient semitransparent organic solar cells." Joule 3.9 (2019): 2241-2254.

7. Bates, Matthew, et al. "High Efficiency Transparent and Semi-Transparent Photovoltaics Based on a Layer-By-Layer Deposition." Solar RRL 7.10 (2023): 2200962.

8. Song, Yu, et al. "Semitransparent organic solar cells enabled by a sequentially deposited bilayer structure." ACS applied materials & interfaces 12.16 (2020): 18473-18481.

9. Xiong, Yuan, et al. "Novel Bimodal Silver Nanowire Network as Top Electrodes for Reproducible and High-Efficiency Semitransparent Organic Photovoltaics." Solar Rrl 4.10 (2020): 2000328.

10. Liu, Yuqiang, et al. "Unraveling sunlight by transparent organic semiconductors toward photovoltaic and photosynthesis." ACS nano 13.2 (2019): 1071-1077.
